# A Semi-Supervised Method for Predicting Transcription Factor–Gene Interactions in *Escherichia coli*

**DOI:** 10.1371/journal.pcbi.1000044

**Published:** 2008-03-28

**Authors:** Jason Ernst, Qasim K. Beg, Krin A. Kay, Gábor Balázsi, Zoltán N. Oltvai, Ziv Bar-Joseph

**Affiliations:** 1 Machine Learning Department, School of Computer Science, Carnegie Mellon University, Pittsburgh, Pennsylvania, United States of America; 2 Department of Pathology, University of Pittsburgh, Pittsburgh, Pennsylvania, United States of America; 3 Department of Systems Biology, University of Texas M. D. Anderson Cancer Center, Houston, Texas, United States of America; Washington University, United States of America

## Abstract

While *Escherichia coli* has one of the most comprehensive datasets of experimentally verified transcriptional regulatory interactions of any organism, it is still far from complete. This presents a problem when trying to combine gene expression and regulatory interactions to model transcriptional regulatory networks. Using the available regulatory interactions to predict new interactions may lead to better coverage and more accurate models. Here, we develop SEREND (SEmi-supervised REgulatory Network Discoverer), a semi-supervised learning method that uses a curated database of verified transcriptional factor–gene interactions, DNA sequence binding motifs, and a compendium of gene expression data in order to make thousands of new predictions about transcription factor–gene interactions, including whether the transcription factor activates or represses the gene. Using genome-wide binding datasets for several transcription factors, we demonstrate that our semi-supervised classification strategy improves the prediction of targets for a given transcription factor. To further demonstrate the utility of our inferred interactions, we generated a new microarray gene expression dataset for the aerobic to anaerobic shift response in *E. coli*. We used our inferred interactions with the verified interactions to reconstruct a dynamic regulatory network for this response. The network reconstructed when using our inferred interactions was better able to correctly identify known regulators and suggested additional activators and repressors as having important roles during the aerobic–anaerobic shift interface.

## Author Summary

The proper functioning of transcriptional gene regulation is essential for all living organisms. Several diseases are associated with loss of appropriate transcriptional regulation. Even in relatively simple organisms, such as the bacterium *E. coli*, response to environmental stress is a complex and highly regulated process. This process is controlled by a set of transcription factors that causes an increase or decrease in the expression levels of their target's gene. However, identifying the set of targets regulated by each of these factors remains a challenge. Even after decades of experimental research on *E. coli,* only a quarter of all gene products have a known regulator. Here, we develop a method that extends the known set of regulator–target relationships with additional predictions. Our method utilizes the DNA sequence control code and expression levels of known targets in a variety of conditions, as well as genes for which it is not known if they are targets of a specific regulator. We show that our method more accurately identifies true targets of known regulators than previous methods suggested for this task. We then applied our predictions to identify active regulators involved in the dynamic response that occurs in *E. coli* when it is deprived of oxygen.

## Introduction

Decades of research on the bacterium *Escherichia coli* have led to the accumulation of a large knowledge base about transcriptional regulation within this prokaryotic model organism. Researchers have electronically encoded in databases (such as EcoCyc and RegulonDB) thousands of activation and repression relationships among transcription factors (TFs) and genes [1]–[3]. However, while *E. coli* has one of the most comprehensive datasets of experimentally verified transcriptional regulatory interactions of any organism, it is still far from complete. For instance, the experimentally verified and curated TF-gene interactions provides regulatory relationships for only approximately 1000 genes, which is well below the more than 4000 genes predicted to be present in *E. coli*. This relatively low coverage of the experimentally verified and curated interaction network presents a challenge when attempting to reconstruct the active regulatory network for a condition of interest based on microarray gene expression data. When analyzing microarray experiments, researchers often need information about the set of genes predicted or known to be regulated by various TFs. This information can then be used to determine the influence of the TFs in the condition of interest by indirectly observing the activity of the regulated genes, even for cases in which the TF is post-transcriptionally regulated [4]–[6].

A traditional computational approach to identify additional gene targets of a TF, which has been applied to *E. coli*, is to characterize the DNA sequence binding preferences of a TF based on an alignment of known binding sites of the TF, and then use this alignment to scan the promoter region of genes for sites matching the preferences [7]. In some cases researchers have used conservation as an additional filter [8]–[10] or extended the alignment based approach using a biophysical based model [11]. While it has been shown that for some TFs in *E. coli* the presence of a motif can be highly predictive of true binding [12], for other TFs the motif pattern is more degenerate leading to reduced accuracy. An additional limitation in *E. coli*, where genes are organized into transcriptional units and many TFs function as both activators and repressors [2], is that motif scanning only determines the binding site location, which is not sufficient to determine if a specific binding site is being used to activate or repress a specific gene [13].

Another approach researchers have taken to predicting TF-gene interactions utilizes just mRNA expression data by evaluating whether the expression level of the TF and the target gene are consistent with a regulatory relationship. Faith et al. [14] surveyed and evaluated a number of these methods using a compendium of *E*. coli gene expression data. They also introduced a new method for this task: The context likelihood of relatedness (CLR) which extends Relevance Networks [15]. CLR was found to be the top performing method by Faith et al. at recovering known interactions. Other methods considered by Faith et al. include ARACNe [16], Bayesian Networks [17] and linear regression networks. The Relevance Network approach directly ranks TF-gene interactions based on a statistical measure such as the correlation coefficient or mutual information of the expression profile pairs. CLR extends Relevance Networks by considering the distribution of values obtained by the statistical measure for all pairs involving the same TF or regulated gene. The authors found in their evaluation that for CLR and Relevance Networks the best results were obtained using mutual information and the square of the correlation coefficient, respectively. As these methods predict network interactions exclusively from expression data this provides the advantage of being broadly applicable to organisms for which prior knowledge on gene regulation is limited. However in the case of *E. coli* these methods are unable to take advantage of known interactions or DNA sequence binding information to improve the accuracy of the predicted interactions. In particular these methods can only identify interactions for factors that are transcriptionally regulated, which may lead to missing many interactions for post-transcriptionally regulated factors.

**Figure 1:**
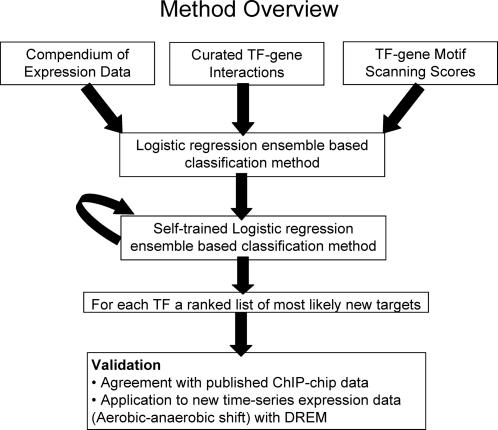
Method overview. SEREND takes as input a compendium of expression data [14], a curated set of *E. coli* TF–gene interactions with direct evidence [1], and scores for TF–gene motif association based on the PWMs present in RegulonDB [2]. SEREND uses a logistic regression ensemble-based classification method where all non-confirmed targets were initially treated as unregulated by the TF. SEREND then relaxed this assumption using a self-training method. We evaluated the ranked predictions of SEREND using published ChIP-chip data, and by combining SEREND's predictions with a new set of time series gene expression data on aerobic-anaerobic shift response in *E. coli*.

In this paper we introduce a new method, SEREND (SEmi-supervised REgulatory Network Discoverer), to predict TF-gene regulatory interactions in *E. coli* (Figure 1). SEREND is an iterative semi-supervised computational prediction method that takes advantage of known regulatory interactions in *E. coli* and extends them by leveraging TF sequence binding affinities and a compendium of expression data. Similar to other methods [4]–[6] SEREND does not assume that a TF is necessarily transcriptionally regulated. Instead SEREND uses expression data in the context of known or predicted TF-gene interactions. However, these previous methods assume a fixed set of TF-gene interactions, while the purpose of SEREND is to predict additional TF-gene interactions. These predictions can later be used as input to these other methods, as we demonstrate for one method on a new expression dataset. Other methods performed iterative analysis as SEREND does here [18],[19]. However, unlike SEREND, which focuses on classification, the goal of these prior methods was clustering or gene set module identification leading to different treatment for the features used and different meanings for the resulting sets. Another method [20] used curated interactions and expression data along with Gene Ontology (GO) and phylogenic similarity to predict additional gene targets, but did not use an iterative or semi-supervised approach or motif information as we do here. We chose for our method not to use GO annotations in generating predictions giving us the advantage of being able to use GO for an unbiased assessment of the functional role of predicted targets.

In evaluating SEREND, we first establish that SEREND can successfully recover many direct gene targets implicated in chromatin immuno-precipitation (ChIP)-chip experiments and compare its ability to do so with other methods. To further test the predictive capability of SEREND and to assess the functional relevance of the newly-predicted TF-gene interactions, we combine them with new temporal microarray gene expression data obtained during the switch from aerobic to anaerobic growth conditions in *E. coli*. For this we use a recently introduced computational method, Dynamic Regulatory Events Miner (DREM) [4], that allows us to analyze and model the dynamics of the transcriptional regulatory network in response to this environmental change. As we show, the reconstructed network response agrees well with known responses during the *E. coli* aerobic-anaerobic switch. Moreover, by using the new TF-gene interactions predicted by SEREND, DREM is also able to suggest additional TFs as controlling different stages of the aerobic-anaerobic switch response in *E. coli*.

## Results

### Ranking New Predictions for a TF

Figure 1 outlines our strategy to generate ranked predictions of additional targets of a TF, including the direction of the interaction (activator or as a repressor). We first extracted from EcoCyc 11.5 all genomic targets of TFs among the 4205 genes that we considered that have been validated by direct experimental evidence (see Materials and Methods). We also extracted the directions of these interactions. This gave us 1760 interactions corresponding to 123 TFs and 974 genes. See Table S1 for the distribution of the number of confirmed targets across TFs. We also obtained the expression value of all the genes across a diverse set of 445 experimental conditions based on a previously assembled compendium including genetic knockout experiments, overexpression experiments, and environmental stress conditions [14]. Finally for 71 of the 123 TFs we obtained a sequence binding affinity matrix from RegulonDB. We used these matrices to determine a score for the maximum agreement of the TF with a potential binding site at the promoter region of each gene (see Material and Methods). For the remaining 52 TFs the motif score was set to a constant 0, but otherwise the method remains the same.

We next used these features to obtain a ranked prediction of new interactions for each TF. Our method, SEREND, would first train two logistic regression classifiers for each TF. The first classifier uses the expression compendium to predict whether a gene is activated by, is repressed by, or is not a target of the TF. A challenge in training such a classifier is that there is no available list of genes which are confirmed not to be targets of the TF (negative information). SEREND initially sets the label for all genes without confirmed evidence in EcoCyc to not being regulated by the TF, though later the method will revisit these assignments. The second classifier uses motif information, specifically the score of the best binding site of the TF for each gene. The motif classifier labels are binary, denoting whether a gene is a target of the TF or not. Initially these labels also correspond to whether or not there is direct evidence in EcoCyc supporting the interaction. These two classifiers are then combined using a third “meta” logistic regression classifier. The reason we had SEREND keep the two sets of features separate initially is because of the large number of expression features, as opposed to the single motif feature. A classifier that directly uses both motif and expression data would likely be vastly emphasizing the expression data, whereas by combining the two classifiers SEREND can learn accurate weights independent of the available features. This approach is similar to ensemble methods such as stacking [21] and mixture of experts [22].

**Figure 2:**
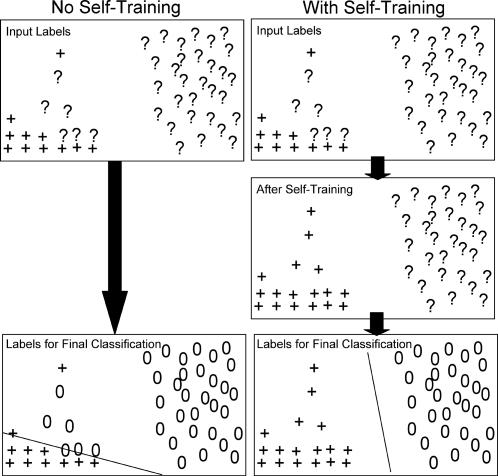
Motivating the self-training method. We abstractly represent the space of expression feature values in two dimensions (though in reality they form a high-dimensional space). The symbol (+) represents an activated target of the TF and the symbol (?) represents genes for which we have no information for this TF. In this example, the ?s on the left side of the rectangles are actually true targets of the TF, while those on the right are not. Without self-training we assume all unknown genes are unregulated by the TF (denoted by “0”) when forming our final classification boundaries. On the right, the self-training procedure would change the labels of some of the unknown genes to being activated targets of the TF before the final classification, which leads to a better classification boundary.

As we noted above, to generate a negative set SEREND used all genes without a direct evidence annotation in EcoCyc. While a vast majority of the genes in this set are indeed not regulated by the TF, some are real targets that have not been discovered to date. We thus had SEREND modify the labels for some of these genes using a type of semi-supervised classification method called self-training [23]. Semi-supervised methods of classification use unlabeled data in conjunction with labeled data to improve classification (Figure 2). The self-training method of SEREND would change the label of genes from not being regulated by a TF to being regulated by the TF if the probability with which the meta-classifier classifies the gene for being regulated by the TF was sufficiently higher than expected (see Materials and Methods). The method then combined these new target predictions with the targets from the previous iteration and used them in a new iteration to re-train a classifier and repeated the process until convergence (no labels changed during an iteration).

On the Supporting Website, we provide for each TF the rank ordering of all genes including activator or repressor prediction labels. In Table 1, we present SEREND's top prediction for the 25 TFs with the most curated targets in our input set. We note that six of these predictions are already curated in EcoCyc based on indirect experimental evidence (this information was not used when training). We also provide in Table 1 brief comments on many of these interactions based on a literature search. In a number of cases we found additional evidence to support the predictions, including in some cases direct evidence that is not presently curated into EcoCyc.

**Table 1:** Top gene predictions.

TF	Gene	Prediction Direction	EcoCyc Indirect	CLR Network	Tractor DB	Comments
CRP	b1498, *ydeN*	1			Yes	Also implicated based on conserved motif analysis in [10]
IHF	b1748, *astC*	1				DNaseI footprinting evidence [58]
Fis	b3864, *spf*	1				ChIP-chip signal peak in promoter region that did not meet stringent threshold [25]
FNR	b1256, *ompW*	1	1		Yes	LacZ reporter with mutant evidence [33]; evidence from microarray expression of mutant [28]
ArcA	b2210, *mqo*	−1				LacZ reporter with mutant evidence [59]
H-NS	b1951, *rcsA*	−1	−1			LacZ reporter with mutant evidence [60]; ChIP-chip evidence [27]
NarL	b1588, *ynfF*	−1			Yes	Evidence from microarray expression data of NarXL mutant [28]
Lrp	b1480, *sra*	−1				Gel shift assay and site-directed mutagenesis evidence confirmed binding, regulates neighboring gene [61]
ModE	b1223, *narK*	1				DNaseI footprinting evidence of binding, but hypothesis binding is used to regulate neighboring gene [43]
CpxR	b2252, *ais*	−1				
ArgR	b0860, *artJ*	−1	−1			Microarray and RTq-PCR expression evidence [62]
FruR	b2168, *fruK*	−1	−1		Yes	Confirmed with direct binding evidence in *Salmonella typhimurium* [63]
NarP	b1224, *narG*	1				
FlhDC	b1070, *flgN*	1	1	Yes		Confirmed with direct binding evidence in *Proteus mirabilis* [64]
IscR	b1901, *araF*	−1				
Fur	b1452, *yncE*	−1			Yes	Evidence from microarray expression of mutant [65]
PurR	b1849, *purT*	−1			Yes	LacZ reporter with mutant evidence [66]
CysB	b2762, *cysH*	1	1			Confirmed with direct binding evidence in *Salmonella typhimurium* [67]
PhoB	b4068, *yjcH*	1				
NagC	b2677, *proV*	−1				
FhlA	b1924, *fliD*	1				
LexA	b1061, *dinI*	−1		Yes	Yes	Gel shift assay and site-directed mutagenesis [68]; ChIP-chip evidence [12]
OxyR	b4367, *fhuF*	1				DNaseI footprinting evidence [69]
SoxS	b2530, *iscS*	1				
GadE	b3506, *slp*	1		Yes		Inferred from microarray expression analysis that gene is either directly regulated by GadE or by YdeO [70]

For each of the 25 TFs with the most curated direct evidence targets, the table shows the top prediction of SEREND of an additional gene target and whether the prediction is that the TF is an activator (“1”) or repressor (“−1”) of the gene. Also noted is whether the interaction is curated into EcoCyc based on indirect evidence, as well as whether the interaction is present in the CLR 60% confidence network [14] or Tractor DB [8]. CLR and Tractor DB do not specify activator or repressor relationships. The last column contains comments about literature evidence supporting the interaction.

### Evaluation of Predictions: Comparison with ChIP-chip Data

We initially focused our evaluation on the ability of methods to recover gene targets implicated in ChIP-chip experiments for five global regulators CRP [24], Fis [25], FNR [26], IHF [25], and H-NS [27]. For each of these we extracted the interactions that are not currently present in the EcoCyc database with direct evidence. As the authors of these papers only reported the genes immediately adjacent to or overlapping the signal peak, we extended their lists to include any gene sharing the same transcriptional unit based on the RegulonDB defined transcriptional units. We note that these sets of genes will not necessarily include all genes regulated by the TF. In some cases these TFs have been reported to bind at many places in the genome with a weaker and more ambiguous signal level than for the lists we are using [24],[25]. In other cases targets of a TF may not be recovered because of condition specific binding or technical limitations of the ChIP-chip protocol [26]. Despite these limitations, we still consider these lists to be a valuable resource for comparing methods aimed at identifying additional direct targets of a TF.

**Figure 3:**
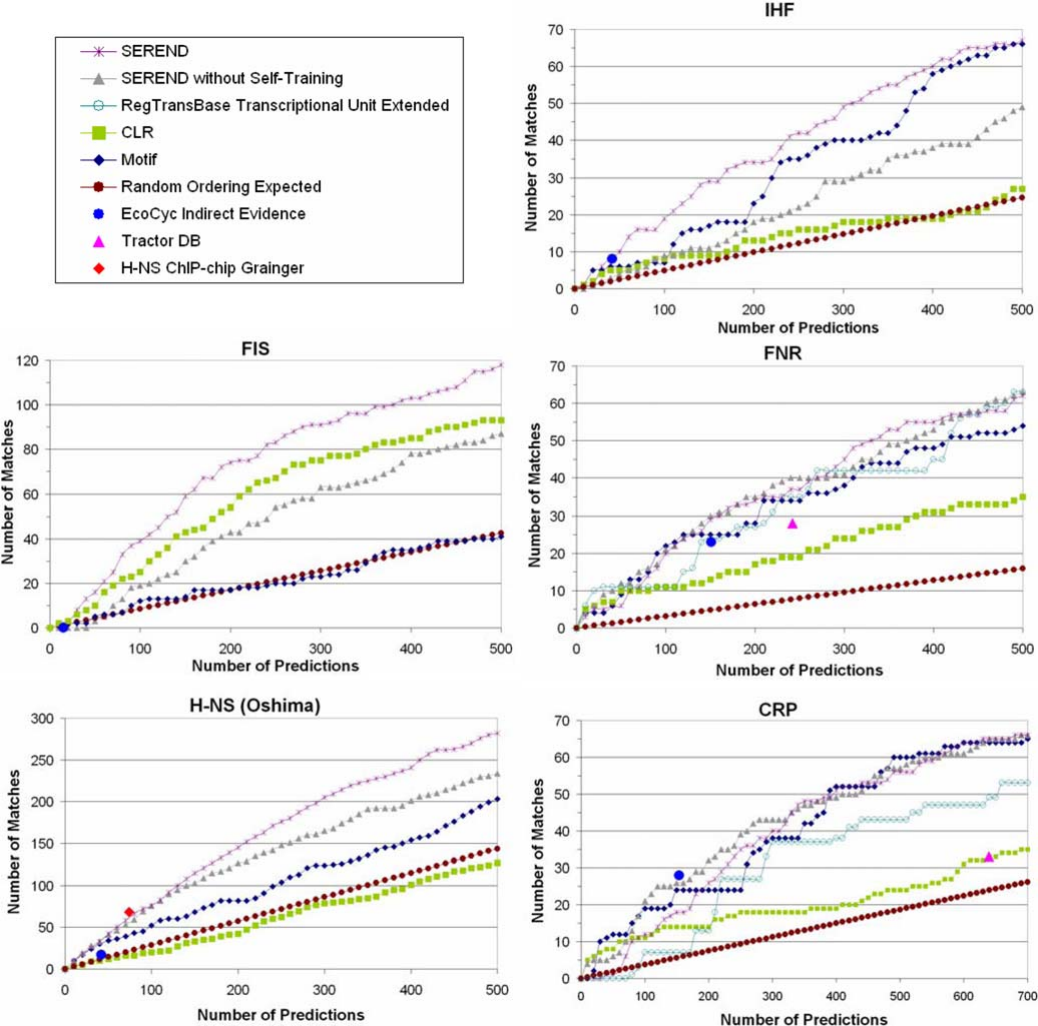
Comparison of methods to predict gene targets implicated in ChIP-chip experiments. The graphs show an evaluation of several methods in terms of predicting targets of the global regulators CRP [24], Fis [25], FNR [26], H-NS [27], and IHF [25] implicated by ChIP-chip experiments, but not curated into the EcoCyc database with direct evidence (see Materials and Methods). We compared SEREND to a version of SEREND without self-training, the CLR method [14], just using our motif values (Motif), and random predictions. We also compare at a single prediction level with the genes curated into EcoCyc from the literature as targets of the TF based on indirect evidence. For CRP and FNR we compare with the Tractor DB predictions [8] and predictions based on RegTransBase [9], and for H-NS with the results of a different ChIP-chip experiment [25]. The *x*-axis represents the number of predictions made by the method (excluding targets already in EcoCyc with direct evidence), and the *y*-axis represents the cumulative number of matches recovered. Note the *x*-axis scale for CRP and the *y*-axis scale for Fis and H-NS are different than the others.

In Figure 3, we plot separately for each TF on the *x*-axis the number of gene predictions a method made up to either 500, or in the case of CRP 700, excluding predictions that already have direct evidence in EcoCyc. On the *y*-axis, we show the number of matches to the set of genes in our ChIP-chip defined gene set, for each number of predictions. We compare the predictions of SEREND to those that would be generated by it if it did not use the self-training procedure. We also compare these results to motif-based predictions and the previously reported predictions of the CLR method with mutual information [14]. As a baseline, we also compare the expected number of matches with a method that simply randomly orders the genes. In each graph, we plot a point representing the number of genes curated in EcoCyc to be a target of the TF based only on indirect evidence (e.g. gene expression data or presence of a binding site motif). For the FNR and CRP graphs we also compare to the Tractor DB method [8] and a prediction ordering we derived based on RegTransBase (see Materials and Methods), both methods use motif and conservation information. Tractor DB did not make any predictions for H-NS, IHF, and only one for Fis, and RegTransBase did not directly support these TFs.

As the charts in Figure 3 show, for Fis, IHF, and H-NS there is a sizeable improvement for SEREND derived from its use of the self-training procedure. For FNR the results of SEREND as compared to a version without the self-training procedure are about the same, and for CRP the version without self-training achieves more matches over the first several hundred predictions. For all TFs joint predictions based on expression and sequence are better than expected from randomly ordering genes. We found the motif scores to be significantly predictive of in-vivo binding for all but one of the TFs we looked at. Unlike the other TFs, for Fis higher motif scores were not associated with higher likelihood of binding. Combining the motif scores with expression data using SEREND led to a clear overall improvement in all cases except for CRP, where the relative performances varies depending on the number of predictions. Predictions based on RegTransBase [9] and the Tractor DB [8] method for identifying motif targets, both of which used conservation information about motifs, did not show overall improvement in recovering genes in the validation sets for FNR and CRP than just using our motif scores for genes, which does not consider motif conservation. Interestingly we note our predictions for H-NS are competitive with the set of targets reported by a second ChIP-chip experiment of [25], indicating that for this TF the quality of our predictions are within the tolerance expected from differences in laboratory experimental protocols and other experimental noise. The plots also indicate that in all cases except for CRP, SEREND either outperforms or is essentially equivalent to the literature curated interactions without direct evidence, and has the added benefit of allowing more flexibility in the number of predictions selected. See the Text S1 for extended versions of these plots including a comparison with Relevance Networks [15] using the square of the correlation coefficient, and knockout experiments for FNR [28].

### Biological Functional Analysis of Predicted Targets of Global Regulators

We used a GO enrichment analysis to characterize the biological functions of newly predicted targets of global regulators and then compared that with an analysis on the set of curated and verified targets. We performed the analysis based on UniProt GO annotations for *E. coli* (see Materials and Methods) for each of the seven TFs with the most targets in EcoCyc (ArcA, CRP, FIS, FNR, H-NS, IHF, and NarL). In Table 2 we list for each TF the top ranked GO category among its predicted targets along with the enrichment p-value, as well as the p-value for this category among the curated targets. We observe that for ArcA, CRP, and FNR the top ranked GO category based on the predicted targets is significant in the analysis on the curated targets, which was not the case for FIS, H-NS, IHF, and NarL. For FIS, the most significant GO category among the new predictions was the structural constituent of ribosome. FIS does have a known role in regulating ribosomal RNA genes [29], and among our newly predicted targets of FIS are a significant number of ribosomal proteins. For H-NS, its involvement in transposition has been previously demonstrated [30]. For IHF, the most significant category was the lipopolysaccharide biosynthetic and metabolic processes. The role of IHF in capsular polysaccharide biosynthesis has been previously discussed [31]. For NarL, the parent category of nickel ion binding in the GO hierarchy, transition metal ion binding, was highly significant among curated genes (p-val <10^−10^). See Text S1 for additional GO categories significant among either the predicted or curated gene sets. These results support the assignments made by SEREND and indicate that the newly predicted targets for most TFs can be used to correctly extend our understanding of the function of these TFs.

**Table 2:** Top GO categories for predicted gene sets.

TF	Top GO Category for Predicted Target	p-Value, Predicted Targets	p-Value, Curated Targets
ArcA	Cellular respiration	2×10^−10^	2×10^−15^
CRP	Carbohydrate transport	3×10^−14^	6×10^−25^
Fis	Structural constituent of ribosome	2×10^−33^	0.84
FNR	4 iron, 4 sulfur cluster binding	4×10^−3^	3×10^−14^
H-NS	Transposition, DNA-mediated	2×10^−4^	0.11
IHF	Lipopolysaccharide biosynthetic/metabolic process	4×10^−11^	1
NarL	Nickel ion binding	3×10^−7^	1

The table shows the most significant GO categories for new predicted gene targets for the TFs, with the most curated targets in EcoCyc. The table compares the enrichment p-value of this category for the newly predicted targets and the curated targets.

### Application to Aerobic–Anaerobic Shift

The above analysis with ChIP-chip data focused on establishing that SEREND's predictions are significantly over-represented within the set of direct binding targets of the TF. We also evaluated whether the gene expression level of SEREND's target predictions are consistent with that of known targets of these TFs. Additionally, we tested if the activator and repressor predictions are accurate for TFs that function in both roles. We performed this evaluation on new temporal microarray gene expression data (Gene Expression Omnibus accession GSE8323) that we generated for the shift from aerobic to anaerobic growth during steady state culture conditions of *E. coli* (see Material and Methods). In this bacterium, in response to the lack of oxygen in the growth medium, two TFs, FNR (fumarate-nitrate reductase regulator) and ArcA TFs (aerobic respiratory control), are known to be the master regulators of this response. FNR is a key regulator of respiration and it controls the transcription of many genes whose functions facilitate adaptation to growth under O_2_-limiting conditions [32]–[36]. Under microaerobic conditions, ArcA induces expression of several gene products of the central carbon metabolism, which are sensitive to lower levels of oxygen, and it represses many genes of aerobic respiration [37]–[39]. NarL and NarP are two other TFs known to be involved in the aerobic-anaerobic shift response, and both of them regulate expression of several operons in response to nitrates and nitrites during anaerobic respiration and fermentation [28],[40],[41]. However, while the roles of the TFs listed above have been well characterized in aerobic-anaerobic response, the identity and roles of some other TFs are less clear.

### Comparison of Predicted and Curated TF–Gene Interactions Using New Expression Data

**Figure 4:**
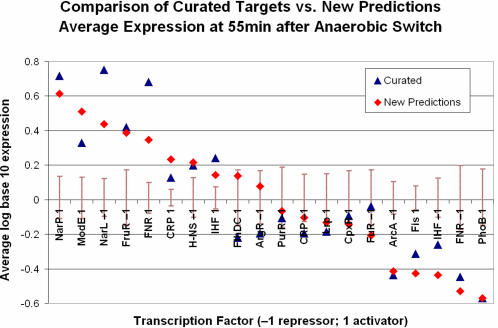
Transcription factor target set agreement between predicted and curated targets. The average expression values for TF regulatory modes (TF and activator or repressor relationship) among curated and new predicted targets at the 55-min time point of the new aerobic–anaerobic shift gene expression data are shown. Only the top 20 TF regulatory modes in terms of the number of new predictions are included. We excluded genes with dual annotations from the curated averages. We included genes in the predicted set averages for which we had a new prediction with regards to the mode of interaction (either because they were dual-annotated or SEREND predicted the opposite mode; this generally was for a small number of genes; see Table S1). For each TF regulatory mode, the graph also displays the 95% confidence interval based on 10,000 random draws of new predicted targets of the same size set. The graph shows that the average expression for a number of predicted TF target gene sets was significantly induced or repressed. The graph also shows a good agreement for most TF target gene sets between the curated and predicted sets, indicating the accuracy of the predictions.

To compare the set of interactions in the curated databases with the new targets predicted by SEREND, we first focused on expression values measured at the last sampled time point, 55 min after the shift from aerobic to anaerobic growth. Since these expression values were not used to generate our predictions they provide an unbiased test set for our predictions. We compared the average expression of the two sets of targets (curated and new predictions) for each TF activity mode (i.e., a factor and its influence as an activator or a repressor). In Figure 4, we plot the average expression of the two sets for the top 20 TF activity modes in terms of the number of new predictions (see Materials and Methods). We also plot a 95% confidence interval based on 10,000 randomizations for selecting sets of the same size as the new predictions (curated predictions confidence intervals were similar). Figure 4 illustrates a good agreement between the average expression of the curated targets and the newly predicted targets for this new expression dataset. We observe that the predicted and curated predictions completely agree on which are the top 8 most significantly upregulated gene sets and which are the top 5 most significantly downregulated gene sets. From Figure 4 we also observe that on average CRP, FNR, and IHF predicted activated targets had an induced expression level, while the predicted repressed targets had a repressed expression level.

### Dynamic Transcriptional Regulatory Map of the Aerobic–Anaerobic Condition

**Figure 5:**
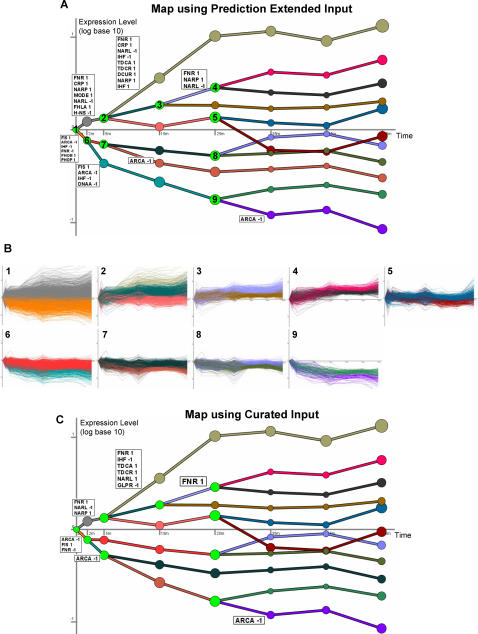
Inferred dynamic regulatory maps of *E. coli* response to the aerobic–anaerobic shift. (A) Dynamic regulatory map inferred by DREM by combining the new aerobic–anaerobic shift microarray gene expression data and our prediction-extended TF–gene interaction dataset. The numbered green nodes represent the split points. DREM assigned genes to their most likely path through the splits. Paths out of the splits are annotated with TF regulatory modes that are associated with genes assigned to the path at a score <10^−4^, and the annotations are ranked ordered using the score (see Text S2). A “1” after the TF symbol denotes activation mode and a “−1” denotes repression mode. The area of a node is proportional to the standard deviation of the expression of the genes traversing through that node. (B) The genes traversing through the nine splits are shown in (A). The number in the upper left of the plot corresponds to the number of the split. Genes are colored based on their path out of the split. (C) The DREM map inferred when using for the TF–gene input only curated interactions with direct evidence.

We next derived an annotated dynamic regulatory map for the *E. coli* aerobic-anaerobic shift response by combining the measured time series expression data with known interactions from EcoCyc that we extended with SEREND's new predictions. We used DREM [4] to derive the regulatory response network. DREM models gene regulation as a cascade of split events controlled by specific TFs. Split events are points in the time series where prior to the split genes have roughly the same expression levels, but after the split have separate expression distributions (Figure 5). By examining the set of genes assigned to different paths going out of a split, DREM labels these paths with the TFs controlling them including whether the TF regulates the genes as an activator or a repressor.

In Figure 5A we number the splits, and then in Figure 5B, we display for each split the corresponding genes assigned to a path originating from the split. The color of the genes in Figure 5B corresponds to the color in Figure 5A of the path out of the split to which DREM assigned them. The map indicates that by 2 min those genes that were eventually upregulated (gray-colored genes), already had a different distribution than those which were downregulated (orange-colored genes). Among GO categories, the upregulated genes were most enriched for carbohydrate transport (p-val <10^−8^), while the downregulated genes were most enriched for biosynthetic process genes (p-val <10^−30^) including translation genes (p-val <10^−24^). The map also indicates that between 5 min and 25 min there was a large change in expression distribution among the genes most activated and repressed in this condition. The last split event in the map occurs 25 min after the response, and the paths remain mostly unchanged thereafter, indicating that by 35 min at the transcriptional level *E. coli* has adapted to the anaerobic conditions. This also suggests that the transitional events that have occurred between 0–35 min after switching to an anaerobic state are events associated with the microaerobic response. The cascade of splits occurring before 25 min of the shift suggests that *E. coli* cells are slowly adapting to the anaerobic conditions during the initial phases of the shift. In Text S1 we further discuss the GO categories enriched in these various splits. DREM has also identified several known and new TFs as regulators of this shift as we discuss below.

### Comparison to Using Only the Curated Network

**Figure 6:**
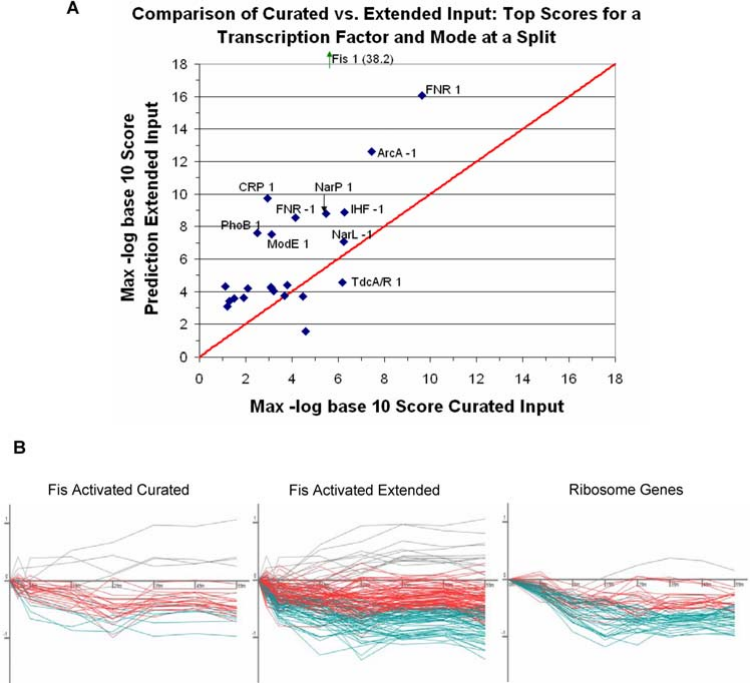
Impact of using prediction-extended TF–gene input to DREM. (A) *x*-axis (*y*-axis) is the maximum of the negative of the log base 10 score of the TF and regulatory mode at any split using the curated TF–gene input (prediction-extended TF–gene input). Any point above the diagonal line received a more significant score using our predictions. As we show in Text S1 using randomization analysis, this is not because we used a larger set of interactions input. The negative log base 10 score for Fis (38.2 using our predictions and 5.7 using the curated EcoCyc list) is not plotted to keep the dimension of the scale reasonable. (B) (Left panel) The expression of non-filtered genes annotated with direct evidence in EcoCyc as being activated by Fis. Color-coding of genes correspond to path assignments between 5 and 10 min in the maps of Figure 5. (Center panel) The genes in the predictions extended network that are annotated as being activated by Fis. (Right panel) All GO-annotated ribosome genes in the dataset meeting the filtering criteria. There is a significant overlap between these genes and Fis-activated genes in the predicted network.

The map of Figure 5A was based on known targets from EcoCyc and extended with our new predictions. To determine if the added predictions improved our ability to reconstruct this regulatory network, we compared this to the map recovered by DREM when using only the curated interactions from EcoCyc with direct evidence. Figure 5C presents the regulatory map identified when using only the curated interaction data as input. While some of the paths share the same annotations in both maps, in the vast majority of cases the score is more significant when using the predicted set. Figure 6A presents a scatter plot of the most significant scores of the TFs (for those with scores lower than 0.001). Reassuringly, we observe a substantial increase in significance for important TFs for this response, such as ArcA, FNR, and NarP. As a control, we considered adding random predictions and found that these did not improve scores but rather decreased them (see Text S1).

An interesting observation is the large increase in significance of the score of Fis activated genes when including the predicted interactions. Furthermore, Fis is seen associated with repressed paths for two splits in Figure 5A, but only the first split in Figure 5C. In the left panel of Figure 6B, we show the expression of those Fis activated genes that are in the curated input. In the center panel of Figure 6B, we show the expression pattern of those Fis activated targets that are in our prediction extended network. On the right panel in Figure 6B, we plot the expression of GO annotated ribosome genes. When using only the curated data, the mechanism by which these ribosomal genes are regulated as part of this response is unexplained, as only three of these genes have a regulator with curated direct evidence. In contrast, when using the new predictions many of these ribosomal genes are determined to be activated by Fis (31 of the 56 genes, p-val<10^−28^). Of these 31 genes, 21 are on the list of genes bound by Fis in [25] or are in the same transcriptional unit as a gene from this list. The potential importance of the effect of Fis in altering the expression of ribosome genes in response to the aerobic-anaerobic shift is something that would have been missed by the method had we not extended the curated network with additional predictions.

## Discussion

A large amount of experimental data has accumulated regarding TF-gene regulatory information for *E. coli*. However, this information is not complete. Many of the genes in *E. coli* do not have any validated regulators and it is likely that many interactions are unknown even for those genes with one or more validated regulators. To make optimal use of the curated information, methods should leverage this information as much as possible when making additional predictions of TF-gene regulatory interactions. Such predictions would then be useful when combined with other high throughput data measuring responses of all *E. coli* genes in a condition of interest.

Here we presented a new semi-supervised learning-based method, SEREND, which uses curated data, sequence motif information, and a compendium of expression data to predict new TF-gene interactions. Using ChIP-chip data, we have shown that semi-supervised learning can improve predictions regarding TF-gene interactions. Using new temporal gene expression data for the aerobic-anaerobic switch response in *E. coli*, we have shown that these predictions can improve the utility of experimentally-verified interactions when reconstructing dynamic response networks. While the resulting networks utilized some of the new predictions these are primarily for TFs involved in this response. If the TF binds the DNA without effect on transcription in this condition these interactions would not be identified in the resulting map.

The resulting regulatory map for the aerobic-anaerobic response summarizes current knowledge and provides new insights into the role of various TFs in the response. The map labels the activators FNR, CRP, NarP, ModE, FhlA, and H-NS, and the repressors NarL and H-NS as associated with the upregulated genes, those assigned to the induced path in the first split. This means that the method predicts these TFs to be major regulators of the response, and likely the first TFs to upregulate expression of various genes when oxygen is removed from the growth medium. As mentioned above FNR, NarL and NarP are well known to be important regulators in this response. FhlA (formate hydrogen-lyase) is a well known transcriptional activator of *hyc* and *hyp* operons in *E. coli*, and the FNR-mediated regulation of *hyp* expression in *E. coli* has also been described [42], which might indicate that FhlA acts synergistically with FNR in regulating some genes during the anaerobic response. Published evidence has suggested that ModE is a secondary transcription activator of the *hyc* and the *nar* operons (encoding genes in response to nitrates and nitrites) [43] and the *dmsABC* operon under conditions of anaerobiosis [44]. The initial repressed pathway includes targets that are associated with activation by Fis, PhoB, and PhoP (indicating decreased activity of these TFs) and repression by FNR and ArcA. Fis is known to play a major role in reconfiguration of *E. coli* cellular processes by up-and down-regulating expression of various genes during changes in growth conditions, and its expression also varies dramatically during cell growth by autoregulation [45],[46]. Additional TFs that are associated with activated genes at later split events include DcuR, TdcA, TdcR, and IHF. CRP has been described to govern the anaerobic transcriptional activation of the Tdc regulators (TdcA and TdcR) [47], which supports our findings that these are secondary responders.

While we have used ChIP-chip data in evaluating predictions for some TFs, overall the number of TFs for which ChIP-chip data are currently available in *E. coli* is limited [12], [24]–[27],[48]. In addition, unlike SEREND, ChIP-chip experiments do not differentiate between activator and repressor relationship. Furthermore SEREND may discover genes regulated by TFs that ChIP-chip experiments would not recover due to condition-specific binding activity or other experimental noise. Finally there could be cases in which a TF binding is detected in a ChIP-chip experiment, but a gene regulated by the TF is not associated with being a target of TF due to the imperfect process of mapping a TF binding location to a set of regulated genes. While motif input is also sensitive to this mapping, the expression input is not, thus in some of these cases SEREND could still predict the interaction.

One avenue for future work is to extend our semi-supervised methodology to also include data from ChIP-chip experiments in generating predictions. In *Saccharomyces cerevisiae*, a global atlas of TF-gene interactions is available based on ChIP-chip data [49], which researchers improved by combining the ChIP-chip data with other evidence sources, such as sequence motif and gene co-expression information [49]–[51]. Another extension is to apply our methodology for inferring TF-gene interactions to additional model organisms. As computational methods for integrating interaction and expression data become increasingly available, we expect that global atlases of TF-gene interactions will become increasingly important resources for experimental biologists to integrate with specific expression experiments.

## Materials and Methods

### Compendium of Microarray Expression Data

We obtained the compendium of mRNA expression data from the Supporting Website of [14]. We used the Robust Multichip Average (RMA) normalization, which was reported to represent the optimal way of normalizing this microarray data from divergent sources among the several major methods considered [14]. We then transformed the data such that each expression value for a gene was the log base two ratio of its expression value with its average expression value over all the experiments. We excluded from the compendium 140 previously purported genes from this dataset that are no longer considered to be true genes in EcoCyc version 11.5, leaving 4205 genes. We also obtained the CLR predictions for these 4205 genes from the Supporting Website of [14]. In the case of the dimer IHF, CLR gives two different scores corresponding to each of the subunits, we mapped this to one score by taking the more significant of the two scores.

### Curated Regulatory Interactions

The curated regulatory interactions including direction of interaction were from EcoCyc 11.5. Only those interactions with the evidence annotations of Site Mutations, Binding of Cellular Extracts, or Binding of Purified Proteins were accepted as direct evidence. In total we used 1760 interactions among 123 TFs and 974 genes.

### Motif Scanning

For the motif scanning we used the *E. coli* K12 genome version U00096.2 sequence. We obtained the TF-binding site positional weight matrices (PWMs) for 71 of the 123 TFs from RegulonDB version 5.7 [2]. The score of a site is the log-ratio of the probability of observing the sequence under a PWM model compared to a background model, which is similar to the approach of [7]. We used a zero order background model, so under both the PWM and background model, the probability of a site is the product of the probability at each position. Under the background model we set the probability of observing a specific nucleotide to its overall proportion in non-coding regions. Under the PWM model, we set the probability of observing a specific nucleotide at a specific position to the ratio of the count for the nucleotide at that position over the total counts at the position in the PWM. We added a pseduo-count to each entry in the matrix equal to the non-coding region background probability of the corresponding nucleotide. For each gene we obtained its RegulonDB transcriptional unit assignment, which is based on either experimental evidence or computational inference. Six genes were not annotated as belonging to any transcriptional unit, and for these we assumed each was the only gene in their respective transcriptional units. We then determined the first gene transcribed in the gene's transcriptional unit, and the location of the start of the coding sequence of the gene from RegulonDB. We then scanned 50 base pairs downstream of the start of the coding sequence and 300 base pairs upstream, on both strands, recording the highest scoring motif hit. If the gene was annotated to belong to multiple transcription units with different first genes we took the value of the highest scoring site in any of the regions. If the highest score site for a gene was below 0 we set the gene's motif score to 0. In the Supporting Results (Text S1) we plot the distribution of the number of maximum scoring sites at each position relative to the start of the coding sequence of the first gene. From this plot we observed a leveling off of the number of maximum scoring sites by 50 base pairs downstream and 300 base pairs upstream.

### SEREND Method-Ranking Predictions for a TF

To generate ranked predictions of gene targets of a TF, SEREND used three logistic regression classifiers: an expression classifier, a sequence motif classifier, and a meta-classifier that combines the output of these other two classifiers. We will first define SEREND's use of logistic regression in general terms and then discuss the specifics of the three classifiers. When discussing terms specific to a classifier we use a superscript ‘*E*’ for the expression classifier, ‘*S*’ for the sequence motif classifier, and ‘*C*’ for the meta-classifier.

#### Logistic regression

Let *N* be the number of genes (for this application *N* = 4205), and *p* be the number of features to the classifier. Let *x_i_* = (*x_i_*_1_,…,*x_ip_*) where *x_ij_* denotes the value of feature *j* for gene *i*. Let *M* be the number of classes, and let *w_im_* denote the weight with which gene *i* is of class *m*. Let *Y_im_* be an indicator variable that gene *i* is of class *m*. We define
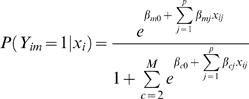
and we set *β_mj_* = 0 for all *j* when *m* = 1. The variables *β_cj_* are determined by maximizing the following function:
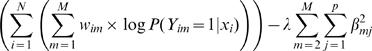
where λ is the regularization parameter, that we selected based on a limited cross-validation analysis. The Weka logistic regression implementation [52] was used to maximize the function above.

#### Expression classifier

For the expression classifier SEREND used 445 features (*p* = 445), and the features for a gene were its value in each of the expression experiments from the compendium [14]. For each TF SEREND considered, the number of classes, *M*, was three, corresponding to a gene being activated by the TF (*m* = 1), repressed by the TF (*m* = 2), or not regulated by the TF (*m* = 3). Let 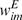 denote the weight with which gene *i* was of class *m*. SEREND initially assumed all genes without direct evidence in EcoCyc [1] were not regulated by the TF, that is 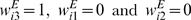. If the gene was only curated with direct evidence to be activated by the TF, then 
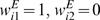
 and 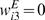. Likewise if the gene was only curated with direct evidence in EcoCyc to be repressed by the TF, then 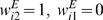 and 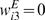. If the gene was curated with direct evidence to be a target of the TF, but not only activated or only repressed by the TF, SEREND set 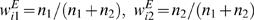, and 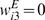 where *n*_1_ and *n*_2_ are the number of genes uniquely annotated to be activated and repressed by the TF respectively (if both *n*_1_ and *n*_2_ were zero, then 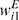 and 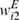 were both initialized to 0.5). λ*^E^* was set to 10.

#### Sequence motif classifier

For the motif classifier there was a single feature (*p* = 1), and this feature represented the maximum agreement of the TF's PWM with a potential binding site in the gene's promoter region based on our motif scanning. The number of classes, *M*, was two with *m* = 1 corresponding to the class that the gene was regulated by the TF and *m* = 2 if the gene was not regulated by the TF. SEREND set 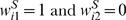 if gene *i* was curated with direct evidence in EcoCyc to be regulated by the TF, without respect to whether the TF functions as an activator or repressor of the gene. If the gene was not in EcoCyc with direct evidence then SEREND set 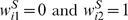. λ*^S^* was set to 1.

#### Meta-classifier

The meta-classifier had two features, (*p* = 2), for a gene *i*. The first feature was the sum of the activated and repressed probabilities with which the expression classifier would classify a gene, that is 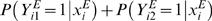. The second feature was the probability the motif classifier gave to the gene for being regulated by the TF, that is 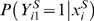. SEREND set 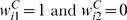 if gene *i* was annotated with direct evidence in EcoCyc to be regulated by the TF, otherwise SEREND set 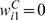 and 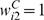. Genes that were not in EcoCyc with direct evidence were ranked by the value 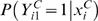. λ*^C^* was set to 1.

#### Self-training procedure

The self-training procedure would change the labels of genes that were previously annotated not to be regulated by the TF to being regulated by the TF if the meta-classifier described above found sufficient evidence that the gene was regulated by the TF. The criterion for re-labeling such a gene was that
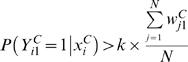
where *k* is a parameter >1 that we set to 2 (see Text S1 for discussion regarding the effects of other values of *k*). To provide justification for this criterion we note that a property of a logistic regression classifier is that the sum of the probabilities for a class equals the count of the observed instances for the class [53] that is we have
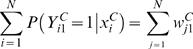
The 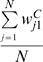 term in the criterion for re-labeling a gene would thus be equal to 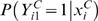 if the probability of being regulated by the TF was uniform across all genes. If the criterion for re-labeling a gene was satisfied, then the classifier gave greater probability than uniform that the gene was regulated by the TF, even though the classifier was trained with the input that the gene was not regulated by the TF. As *k* increases, the greater the probability as compared to uniform would be needed to re-label the gene. If the criterion was met to re-label a gene as being a target of a TF then SEREND set 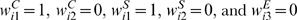. Also for all genes for which 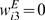, at the start of the iteration or after the relabeling, SEREND set 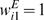 and 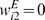 if 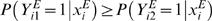 otherwise SEREND set 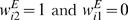. Note that this step specifies a prediction of the more likely direction of interaction for dual instances, and can change the direction for a curated target if inconsistent with other curated targets of the same direction (this occurred for only a relatively small percentage of genes, see Table S1). The method terminates when no change was made to any *w_im_* for any of the classifiers. At no point in this procedure was a gene label changed from being regulated by the TF to not being regulated by the TF. Again the genes that are not in EcoCyc with direct evidence were ranked by the value 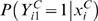.

### Combining Predictions Across TFs

In forming the prediction extended network used in the GO enrichment analysis of global regulators and for the aerobic-anaerobic application, we chose to double the size of the curated network by simply taking for each TF the same number predictions as there were confirmed targets of the TF in the input.

### ChIP-chip Validation Sets

We obtained the list of ChIP-chip implicated target genes for CRP from the Supplement of [24], for Fis and IHF from the Supplement of. [25], for FNR from Table 2 of [26], and for H-NS from the Supplement of [27]. As the authors generally only reported the gene(s) immediately adjacent or covering a signal peak, we extended their lists to include any gene sharing the same transcriptional unit based on the RegulonDB defined transcriptional units. The ChIP-chip implicated target genes we associated with each of these TFs can be found on our Supporting Website. In our evaluation, we excluded genes already confirmed based on direct evidence curated into EcoCycDB to be a target of the TF and genes not in the set of 4205 that we considered. The total number of gene targets in these sets for CRP was 148, for Fis was 347, for IHF was 199, for FNR was 131, and for H-NS was 1191. For H-NS, there is another list of ChIP-chip based targets [25] separate from those of [27]. We chose here to use the list of [27] as the validation set, as it is larger and includes the majority of targets with curated direct evidence, while at the cutoff at which the list of [25] was derived it includes only one curated direct evidence target. We did use predictions based on [25] in the comparison of methods to identify H-NS targets implicated based on [27] (see also Text S1 for the predictions extended by transcriptional units).

### RegTransBase Predictions

We generated ranked predictions for a TF in RegTransBase [9] based on the set of predicted genes returned in the TransTableView for *E. coli* K12 using the default setting for sensitivity on the site score, and specifying to measure conservation based on all genomes for the species *E. coli*. We ranked all genes returned by RegTransBase, meaning the gene had one or more binding sites within 400 basepairs upstream or 50 base pairs down stream of the start of the gene satisfying the sensitivity threshold, based on the maximum conservation score for a site returned for the gene. We then extended the ranked list to include all genes in the same transcriptional unit as listed in RegulonDB. When extended for transcriptional unit a gene received the same site and conservation score, as the highest ranking gene from its transcriptional unit from the original ranked list. A version of the RegTransBase predictions without extending for transcriptional units can be found in the supplement, but did not perform as well.

### Tractor DB–Based Predictions

Predictions for the Tractor DB method [8] were obtained from http://www.ccg.unam.mx/Computational_Genomics/tractorDB/ and http://regulondb.ccg.unam.mx/data/BindingSitePredictionSet.txt. The few predictions that were unique to either of these lists were still used.

### Dynamic Regulatory Maps

We used the Dynamic Regulatory Events Miner (DREM) [4] to reconstruct dynamic regulatory maps of the aerobic-anaerobic shift based on gene expression data and TF-gene association data. Expression values were converted to a log base ten ratio relative to the 0 min time point. We selected only genes with no more than two missing time points and a log base ten fold change of at least 0.3 at one time point, resulting in a total of 2317 genes. The TF-gene association data were a matrix of TFs and genes with an entry being ‘1’ if the TF was predicted to be an activator for the gene, ‘−1’ if it was predicted to be a repressor, and ‘0’ otherwise. Dual regulated genes of a TF in the curated network received the majority label between ‘1’ and ‘−1’ of the other genes regulated by the TF. DREM uses an Input-Output Hidden Markov Model [54] that allows TF-gene interaction information to influence transition probabilities in a gene-specific manner. DREM assigns each gene to its most likely path through the model based on its expression and the TFs that control it. A TF label is assigned to a path out of a split only if based on a hypergeometric distribution calculation its association score with regulating genes along the path out of the split, where a lower score indicates a stronger association, is below a certain cutoff. Here we use 10^−4^ as the cutoff (see Text S1 for maps with other cut-off scores). We used the DREM method as described in [4] except for a change in the model selection criteria. Instead of using a held out test set to evaluate models, under the modified criteria DREM would select models to maximize the log-likelihood minus a regularization penalty on the number of states. This allowed a more explicit penalization of the complexity of the model and allowed DREM to use all data in estimating the parameters and for model selection. See Text S2 for additional details.

### Gene Ontology Enrichment Analysis

The Gene Ontology (GO) enrichment analysis was conducted using STEM [55]. The *E. coli* K12 UniProt GO annotations were obtained from the European Bioinformatics Institute (EBI) at http://www.ebi.ac.uk/GOA/proteomes.html. The reported p-values are uncorrected p-values computed using the hypergeometric distribution; corrected p-values for multiple hypothesis testing appear in Text S1.

### Chemostat Growth Experiment

The *E. coli* K12 strain MG1655 (F^−^ λ^−^ *ilv*G *rfb*50 *rph*1) [56] was grown in a continuous culture using Luria-broth (1-L working volume) in a 2-L bioreactor (BIOFLO 2000, New Brunswick, NJ) under aerobic conditions (45% dissolved O_2_). Once the cells were growing in a steady state (A_600 nm_∼2.5), we collected two 10-ml samples from the culture. After collection of these samples, the growth medium was flushed with N_2_-gas to create anaerobic conditions in the bioreactor. We collected three samples (0, 2, and 5 min) during the immediate transition period. After this, samples were collected at 15, 25, 35, 45 and 55 minutes.

### RNA Isolation, Reverse Transcription, and Microarray Hybridization

The collected cell-culture samples were immediately mixed with 10% of ice-cold stop solution (5% phenol in absolute ethanol) to prevent any additional transcriptional activity, followed by centrifugation at 6,000×*g* for 10 min. The cell pellets were stored at −80°C until further use. The RNA was isolated using EpiCentre's Master Pure RNA isolation kit (Madison, WI) according to manufacturer's protocol. The contaminant DNA was removed by DNase I at 37° C treatment for 30–60 min. The RNA was reverse transcribed into cDNA, which was then used for microarray hybridization on Gene TAC hybridization station (Genomic Solutions), as previously described [56].

### Microarray Analysis

We scanned images from the completed hybridization using a GenePix 4000B array scanner (Molecular Devices, Union City, CA). Raw data were generated using GenePix Pro 3.0 software. Two-color cDNA microarray data are never devoid of spurious technical contributions that originate during array printing, as well as during the collection and processing of samples, fluorescent labeling and hybridization and scanning of the microarray images [57]. To minimize the effect of such contributions, microarray data were normalized, as described before [56] (see also Text S2).

### Supporting Website

The URL for the Supporting Website for this paper is http://www.sb.cs.cmu.edu/ecoli.

## Supporting Information

Text S1 Supporting Results (3.41 MB PDF)

Text S2 Supporting Methods (0.19 MB PDF)

Table S1 Transcription Factors Included in the Study For each transcription factor, the table contains information including whether a motif was available for it, the total number of curated direct evidence targets (the number of predicted targets was the same), and the distribution of activator and repressor targets among these curated and predicted targets.
(0.03 MB XLS)
