# Supplementary material for: A Semi-Supervised Method for Predicting Transcription Factor–Gene Interactions in Escherichia coli
Source: PLoS Comput Biol. 2008 Mar 28;4(3):e1000044. doi: 10.1371/journal.pcbi.1000044 (PMC2266799; doi:10.1371/journal.pcbi.1000044)
Supplement: Text S1 Supporting Results — (3.41 MB PDF) [file pcbi.1000044.s001.pdf]

# Supporting Results for:

# A Semi-Supervised Method for Predicting Transcription Factor-Gene Interactions in *Escherichia coli*

Jason Ernst[1], Qasim K. Beg[2,3], Krin A. Kay[2], Gábor Balázsi[4], Zoltán N. Oltvai[2], Ziv Bar-Joseph[1]

[1]Machine Learning Department, School of Computer Science, Carnegie Mellon University, Pittsburgh, PA, 15213, USA

[2]Department of Pathology, University of Pittsburgh, Pittsburgh, PA, 15261, USA

[4]Department of Systems Biology, University of Texas M. D. Anderson Cancer Center, Houston, TX, 77054, USA

[3]Current Address: Department of Biomedical Engineering, Boston University, Boston, MA, 02215, USA

## Contents

1. GO Analysis of Targets of Global Regulators p2-11

2. GO Analysis Results of Splits in DREM Map p12-15

3. Additional Dynamic Regulatory Maps at Different Score Thresholds p16-17

4. Motif Scanning Distribution of Location of Sites p18

5. Extended Analysis of ChIP-chip Validation p19-20

6. Effect of Self-Training Parameter *k* p21-22

7. Effect of Randomly Extending Curated Network p23-25

References: p26-27

# 1. GO Analysis of Targets of Global Regulators

In this section we present significant Gene Ontology (GO) categories for the seven transcription factors (TFs) with the largest number of predicted and curated targets. The analysis was done separately on the predicted set and the curated set. The p-value was computed based on the hypergeometric distribution. Only categories with at least 5 genes were included in the analysis. The corrected p-value for testing multiple categories is based on a randomization procedure in which a set of the same size was randomly drawn 500 times from the base set of all genes considered. The corrected p-value is then the proportion of times in which a GO p-value for a random set was more significant than an actual p-value. We observe that in some cases a significant GO category for the predicted targets of a TF is also significant for the curated targets, while in other cases the category is only significant among the predicted targets.

ArcA – predicted

| Category ID | Category Name | #Genes Category | #Genes Assigned | #Genes Expected | #Genes Enriched | p-value | Corrected p-value |
|---|---|---|---|---|---|---|---|
| GO:0045333 | cellular respiration | 30 | 9.0 | 0.4 | +8.6 | 1.5E-10 | <0.001 |
| GO:0044248 | cellular catabolic process | 154 | 15.0 | 2.2 | +12.8 | 1.9E-9 | <0.001 |
| GO:0015980 | energy derivation by oxidation of organic com... | 43 | 9.0 | 0.6 | +8.4 | 5.1E-9 | <0.001 |
| GO:0009056 | catabolic process | 169 | 15.0 | 2.4 | +12.6 | 6.8E-9 | <0.001 |
| GO:0009109 | coenzyme catabolic process | 22 | 6.0 | 0.3 | +5.7 | 4.1E-7 | <0.001 |
| GO:0051187 | cofactor catabolic process | 22 | 6.0 | 0.3 | +5.7 | 4.1E-7 | <0.001 |
| GO:0046356 | acetyl-CoA catabolic process | 22 | 6.0 | 0.3 | +5.7 | 4.1E-7 | <0.001 |
| GO:0006099 | tricarboxylic acid cycle | 22 | 6.0 | 0.3 | +5.7 | 4.1E-7 | <0.001 |
| GO:0009060 | aerobic respiration | 23 | 6.0 | 0.3 | +5.7 | 5.5E-7 | <0.001 |
| GO:0006084 | acetyl-CoA metabolic process | 25 | 6.0 | 0.4 | +5.6 | 9.4E-7 | <0.001 |
| GO:0009063 | amino acid catabolic process | 15 | 5.0 | 0.2 | +4.8 | 1.3E-6 | <0.001 |
| GO:0009310 | amine catabolic process | 17 | 5.0 | 0.2 | +4.8 | 2.7E-6 | <0.001 |
| GO:0044270 | nitrogen compound catabolic process | 17 | 5.0 | 0.2 | +4.8 | 2.7E-6 | <0.001 |
| GO:0016491 | oxidoreductase activity | 412 | 15.0 | 5.9 | +9.1 | 4.8E-4 | 0.030 |
| GO:0006732 | coenzyme metabolic process | 113 | 7.0 | 1.6 | +5.4 | 1.0E-3 | 0.054 |
| GO:0006091 | generation of precursor metabolites and ene... | 276 | 11.0 | 3.9 | +7.1 | 1.5E-3 | 0.082 |
| GO:0051186 | cofactor metabolic process | 157 | 7.0 | 2.2 | +4.8 | 6.5E-3 | 0.272 |
| GO:0044262 | cellular carbohydrate metabolic process | 249 | 9.0 | 3.6 | +5.4 | 8.0E-3 | 0.314 |
| GO:0051539 | 4 iron, 4 sulfur cluster binding | 93 | 5.0 | 1.3 | +3.7 | 9.9E-3 | 0.382 |
| GO:0005506 | iron ion binding | 219 | 8.0 | 3.1 | +4.9 | 0.01 | 0.416 |

## ArcA – curated

| Category ID | Category Name | #Genes Category | #Genes Assigned | #Genes Expected | #Genes Enriched | p-value | Corrected p-value |
|---|---|---|---|---|---|---|---|
| GO:0006091 | generation of precursor metabolites and ene... | 276 | 34.0 | 3.9 | +30.1 | 1.3E-25 | <0.001 |
| GO:0048038 | quinone binding | 14 | 13.0 | 0.2 | +12.8 | 3.5E-24 | <0.001 |
| GO:0016491 | oxidoreductase activity | 412 | 35.0 | 5.9 | +29.1 | 6.6E-21 | <0.001 |
| GO:0050136 | NADH dehydrogenase (quinone) activity | 24 | 13.0 | 0.3 | +12.7 | 5.7E-19 | <0.001 |
| GO:0006118 | electron transport | 236 | 27.0 | 3.4 | +23.6 | 6.7E-19 | <0.001 |
| GO:0016655 | oxidoreductase activity, acting on NADH or N... | 25 | 13.0 | 0.4 | +12.6 | 1.2E-18 | <0.001 |
| GO:0003954 | NADH dehydrogenase activity | 25 | 13.0 | 0.4 | +12.6 | 1.2E-18 | <0.001 |
| GO:0009060 | aerobic respiration | 23 | 12.0 | 0.3 | +11.7 | 2.7E-17 | <0.001 |
| GO:0016651 | oxidoreductase activity, acting on NADH or N... | 33 | 13.0 | 0.5 | +12.5 | 1.2E-16 | <0.001 |
| GO:0048037 | cofactor binding | 201 | 23.0 | 2.9 | +20.1 | 6.1E-16 | <0.001 |
| GO:0009109 | coenzyme catabolic process | 22 | 11.0 | 0.3 | +10.7 | 1.2E-15 | <0.001 |
| GO:0051187 | cofactor catabolic process | 22 | 11.0 | 0.3 | +10.7 | 1.2E-15 | <0.001 |
| GO:0046356 | acetyl-CoA catabolic process | 22 | 11.0 | 0.3 | +10.7 | 1.2E-15 | <0.001 |
| GO:0006099 | tricarboxylic acid cycle | 22 | 11.0 | 0.3 | +10.7 | 1.2E-15 | <0.001 |
| GO:0045333 | cellular respiration | 30 | 12.0 | 0.4 | +11.6 | 1.6E-15 | <0.001 |
| GO:0006084 | acetyl-CoA metabolic process | 25 | 11.0 | 0.4 | +10.6 | 7.3E-15 | <0.001 |
| GO:0042773 | ATP synthesis coupled electron transport | 21 | 10.0 | 0.3 | +9.7 | 5.0E-14 | <0.001 |
| GO:0008137 | NADH dehydrogenase (ubiquinone) activity | 21 | 10.0 | 0.3 | +9.7 | 5.0E-14 | <0.001 |
| GO:0015980 | energy derivation by oxidation of organic com... | 43 | 12.0 | 0.6 | +11.4 | 2.5E-13 | <0.001 |
| GO:0009055 | electron carrier activity | 102 | 15.0 | 1.5 | +13.5 | 4.5E-12 | <0.001 |
| GO:0006119 | oxidative phosphorylation | 31 | 10.0 | 0.4 | +9.6 | 5.6E-12 | <0.001 |
| GO:0009056 | catabolic process | 169 | 17.0 | 2.4 | +14.6 | 7.2E-11 | <0.001 |
| GO:0006732 | coenzyme metabolic process | 113 | 13.0 | 1.6 | +11.4 | 3.4E-9 | <0.001 |
| GO:0006120 | mitochondrial electron transport, NADH to ub... | 13 | 6.0 | 0.2 | +5.8 | 1.0E-8 | <0.001 |
| GO:0042775 | organelle ATP synthesis coupled electron tra... | 13 | 6.0 | 0.2 | +5.8 | 1.0E-8 | <0.001 |
| GO:0044248 | cellular catabolic process | 154 | 14.0 | 2.2 | +11.8 | 1.7E-8 | <0.001 |
| GO:0051186 | cofactor metabolic process | 157 | 14.0 | 2.2 | +11.8 | 2.2E-8 | <0.001 |
| GO:0050662 | coenzyme binding | 132 | 13.0 | 1.9 | +11.1 | 2.3E-8 | <0.001 |
| GO:0016310 | phosphorylation | 75 | 10.0 | 1.1 | +8.9 | 6.5E-8 | <0.001 |
| GO:0006796 | phosphate metabolic process | 80 | 10.0 | 1.1 | +8.9 | 1.2E-7 | <0.001 |
| GO:0006793 | phosphorus metabolic process | 81 | 10.0 | 1.2 | +8.8 | 1.4E-7 | <0.001 |
| GO:0044237 | cellular metabolic process | 1627 | 41.0 | 23.2 | +17.8 | 2.7E-6 | <0.001 |
| GO:0044262 | cellular carbohydrate metabolic process | 249 | 14.0 | 3.6 | +10.4 | 6.7E-6 | <0.001 |
| GO:0006631 | fatty acid metabolic process | 35 | 6.0 | 0.5 | +5.5 | 7.7E-6 | <0.001 |
| GO:0032787 | monocarboxylic acid metabolic process | 92 | 8.0 | 1.3 | +6.7 | 3.9E-5 | <0.001 |
| GO:0016614 | oxidoreductase activity, acting on CH-OH gro... | 73 | 7.0 | 1.0 | +6.0 | 6.6E-5 | 0.008 |
| GO:0044464 | cell part | 2139 | 45.0 | 30.5 | +14.5 | 1.0E-4 | 0.010 |
| GO:0016020 | membrane | 1134 | 30.0 | 16.2 | +13.8 | 1.1E-4 | 0.010 |
| GO:0005506 | iron ion binding | 219 | 11.0 | 3.1 | +7.9 | 2.1E-4 | 0.016 |

## CRP – predicted

| Category ID | Category Name | #Genes Category | #Genes Assigned | #Genes Expected | #Genes Enriched | p-value | Corrected p-value |
|---|---|---|---|---|---|---|---|
| GO:0008643 | carbohydrate transport | 107 | 31.0 | 6.4 | +24.6 | 2.9E-14 | <0.001 |
| GO:0005351 | sugar:hydrogen ion symporter activity | 106 | 29.0 | 6.4 | +22.6 | 1.1E-12 | <0.001 |
| GO:0015144 | carbohydrate transmembrane transporter ac... | 106 | 29.0 | 6.4 | +22.6 | 1.1E-12 | <0.001 |
| GO:0051119 | sugar transmembrane transporter activity | 106 | 29.0 | 6.4 | +22.6 | 1.1E-12 | <0.001 |
| GO:0015293 | symporter activity | 135 | 31.0 | 8.1 | +22.9 | 2.6E-11 | <0.001 |
| GO:0009401 | phosphoenolpyruvate-dependent sugar pho... | 57 | 20.0 | 3.4 | +16.6 | 2.8E-11 | <0.001 |
| GO:0019751 | polyol metabolic process | 24 | 12.0 | 1.4 | +10.6 | 2.4E-9 | <0.001 |
| GO:0015291 | secondary active transmembrane transporte... | 166 | 31.0 | 9.9 | +21.1 | 6.4E-9 | <0.001 |
| GO:0006066 | alcohol metabolic process | 124 | 26.0 | 7.4 | +18.6 | 9.5E-9 | <0.001 |
| GO:0009065 | glutamine family amino acid catabolic proce... | 7 | 6.0 | 0.4 | +5.6 | 2.9E-7 | <0.001 |
| GO:0006059 | hexitol metabolic process | 7 | 6.0 | 0.4 | +5.6 | 2.9E-7 | <0.001 |
| GO:0019400 | alditol metabolic process | 7 | 6.0 | 0.4 | +5.6 | 2.9E-7 | <0.001 |
| GO:0019402 | galactitol metabolic process | 7 | 6.0 | 0.4 | +5.6 | 2.9E-7 | <0.001 |
| GO:0022891 | substrate-specific transmembrane transport... | 266 | 37.0 | 15.9 | +21.1 | 6.8E-7 | <0.001 |
| GO:0019544 | arginine catabolic process to glutamate | 5 | 5.0 | 0.3 | +4.7 | 7.4E-7 | <0.001 |
| GO:0006536 | glutamate metabolic process | 12 | 7.0 | 0.7 | +6.3 | 1.6E-6 | <0.001 |
| GO:0022804 | active transmembrane transporter activity | 220 | 32.0 | 13.2 | +18.8 | 1.6E-6 | <0.001 |
| GO:0022857 | transmembrane transporter activity | 310 | 40.0 | 18.6 | +21.4 | 1.7E-6 | <0.001 |
| GO:0006527 | arginine catabolic process | 6 | 5.0 | 0.4 | +4.6 | 4.2E-6 | 0.002 |
| GO:0051234 | establishment of localization | 782 | 75.0 | 46.9 | +28.1 | 6.2E-6 | 0.002 |
| GO:0006810 | transport | 772 | 74.0 | 46.3 | +27.7 | 7.5E-6 | 0.006 |
| GO:0007154 | cell communication | 172 | 26.0 | 10.3 | +15.7 | 7.9E-6 | 0.006 |
| GO:0022892 | substrate-specific transporter activity | 324 | 39.0 | 19.4 | +19.6 | 1.3E-5 | 0.006 |
| GO:0016616 | oxidoreductase activity, acting on the CH-OH ... | 65 | 14.0 | 3.9 | +10.1 | 2.1E-5 | 0.008 |
| GO:0007165 | signal transduction | 150 | 23.0 | 9.0 | +14.0 | 2.2E-5 | 0.008 |

## CRP – curated

| Category ID | Category Name | #Genes Category | #Genes Assigned | #Genes Expected | #Genes Enriched | p-value | Corrected p-value |
|---|---|---|---|---|---|---|---|
| GO:0008643 | carbohydrate transport | 107 | 42.0 | 6.4 | +35.6 | 5.8E-25 | <0.001 |
| GO:0015144 | carbohydrate transmembrane transporter ac... | 106 | 41.0 | 6.4 | +34.6 | 4.5E-24 | <0.001 |
| GO:0051119 | sugar transmembrane transporter activity | 106 | 41.0 | 6.4 | +34.6 | 4.5E-24 | <0.001 |
| GO:0005351 | sugar:hydrogen ion symporter activity | 106 | 41.0 | 6.4 | +34.6 | 4.5E-24 | <0.001 |
| GO:0015293 | symporter activity | 135 | 44.0 | 8.1 | +35.9 | 2.9E-22 | <0.001 |
| GO:0015291 | secondary active transmembrane transporte... | 166 | 45.0 | 9.9 | +35.1 | 3.9E-19 | <0.001 |
| GO:0044248 | cellular catabolic process | 154 | 41.0 | 9.2 | +31.8 | 3.7E-17 | <0.001 |
| GO:0006066 | alcohol metabolic process | 124 | 36.0 | 7.4 | +28.6 | 1.8E-16 | <0.001 |
| GO:0009056 | catabolic process | 169 | 41.0 | 10.1 | +30.9 | 1.4E-15 | <0.001 |
| GO:0022804 | active transmembrane transporter activity | 220 | 45.0 | 13.2 | +31.8 | 4.4E-14 | <0.001 |
| GO:0005975 | carbohydrate metabolic process | 354 | 59.0 | 21.2 | +37.8 | 4.6E-14 | <0.001 |
| GO:0022891 | substrate-specific transmembrane transport... | 266 | 47.0 | 15.9 | +31.1 | 3.4E-12 | <0.001 |
| GO:0005996 | monosaccharide metabolic process | 90 | 26.0 | 5.4 | +20.6 | 4.4E-12 | <0.001 |
| GO:0022857 | transmembrane transporter activity | 310 | 50.0 | 18.6 | +31.4 | 2.2E-11 | <0.001 |
| GO:0009401 | phosphoenolpyruvate-dependent sugar pho... | 57 | 19.0 | 3.4 | +15.6 | 2.5E-10 | <0.001 |
| GO:0044262 | cellular carbohydrate metabolic process | 249 | 42.0 | 14.9 | +27.1 | 2.7E-10 | <0.001 |
| GO:0022892 | substrate-specific transporter activity | 324 | 48.0 | 19.4 | +28.6 | 1.3E-9 | <0.001 |
| GO:0019318 | hexose metabolic process | 80 | 21.0 | 4.8 | +16.2 | 3.9E-9 | <0.001 |
| GO:0019439 | aromatic compound catabolic process | 15 | 8.0 | 0.9 | +7.1 | 6.6E-7 | <0.001 |
| GO:0019299 | rhamnose metabolic process | 5 | 5.0 | 0.3 | +4.7 | 7.4E-7 | <0.001 |
| GO:0019323 | pentose catabolic process | 5 | 5.0 | 0.3 | +4.7 | 7.4E-7 | <0.001 |
| GO:0019566 | arabinose metabolic process | 5 | 5.0 | 0.3 | +4.7 | 7.4E-7 | <0.001 |
| GO:0019568 | arabinose catabolic process | 5 | 5.0 | 0.3 | +4.7 | 7.4E-7 | <0.001 |
| GO:0045893 | positive regulation of transcription, DNA-dep... | 5 | 5.0 | 0.3 | +4.7 | 7.4E-7 | <0.001 |
| GO:0044237 | cellular metabolic process | 1627 | 134.0 | 97.5 | +36.5 | 1.1E-6 | <0.001 |
| GO:0031325 | positive regulation of cellular metabolic proc... | 8 | 6.0 | 0.5 | +5.5 | 1.1E-6 | <0.001 |
| GO:0048518 | positive regulation of biological process | 8 | 6.0 | 0.5 | +5.5 | 1.1E-6 | <0.001 |
| GO:0048522 | positive regulation of cellular process | 8 | 6.0 | 0.5 | +5.5 | 1.1E-6 | <0.001 |
| GO:0009893 | positive regulation of metabolic process | 8 | 6.0 | 0.5 | +5.5 | 1.1E-6 | <0.001 |
| GO:0045935 | positive regulation of nucleobase, nucleosid... | 8 | 6.0 | 0.5 | +5.5 | 1.1E-6 | <0.001 |
| GO:0045941 | positive regulation of transcription | 8 | 6.0 | 0.5 | +5.5 | 1.1E-6 | <0.001 |
| GO:0046164 | alcohol catabolic process | 47 | 13.0 | 2.8 | +10.2 | 2.1E-6 | <0.001 |
| GO:0009109 | coenzyme catabolic process | 22 | 9.0 | 1.3 | +7.7 | 2.2E-6 | <0.001 |
| GO:0006099 | tricarboxylic acid cycle | 22 | 9.0 | 1.3 | +7.7 | 2.2E-6 | <0.001 |
| GO:0051187 | cofactor catabolic process | 22 | 9.0 | 1.3 | +7.7 | 2.2E-6 | <0.001 |
| GO:0046356 | acetyl-CoA catabolic process | 22 | 9.0 | 1.3 | +7.7 | 2.2E-6 | <0.001 |
| GO:0006071 | glycerol metabolic process | 17 | 8.0 | 1.0 | +7.0 | 2.3E-6 | <0.001 |
| GO:0009060 | aerobic respiration | 23 | 9.0 | 1.4 | +7.6 | 3.4E-6 | 0.002 |
| GO:0044238 | primary metabolic process | 1500 | 124.0 | 89.9 | +34.1 | 3.8E-6 | 0.002 |

## Fis – predicted

| Category ID | Category Name | #Genes Category | #Genes Assigned | #Genes Expected | #Genes Enriched | p-value | Corrected p-value |
|---|---|---|---|---|---|---|---|
| GO:0003735 | structural constituent of ribosome | 56 | 31.0 | 1.8 | +29.2 | 2.4E-33 | <0.001 |
| GO:0005840 | ribosome | 57 | 31.0 | 1.8 | +29.2 | 5.2E-33 | <0.001 |
| GO:0030529 | ribonucleoprotein complex | 60 | 31.0 | 1.9 | +29.1 | 4.5E-32 | <0.001 |
| GO:0044444 | cytoplasmic part | 85 | 34.0 | 2.7 | +31.3 | 2.1E-30 | <0.001 |
| GO:0006412 | translation | 111 | 37.0 | 3.5 | +33.5 | 1.0E-29 | <0.001 |
| GO:0043228 | non-membrane-bound organelle | 79 | 32.0 | 2.5 | +29.5 | 8.3E-29 | <0.001 |
| GO:0043232 | intracellular non-membrane-bound organelle | 79 | 32.0 | 2.5 | +29.5 | 8.3E-29 | <0.001 |
| GO:0043229 | intracellular organelle | 96 | 34.0 | 3.0 | +31.0 | 2.8E-28 | <0.001 |
| GO:0019843 | rRNA binding | 37 | 24.0 | 1.2 | +22.8 | 3.0E-28 | <0.001 |
| GO:0009059 | macromolecule biosynthetic process | 256 | 48.0 | 8.1 | +39.9 | 3.0E-26 | <0.001 |
| GO:0003723 | RNA binding | 111 | 33.0 | 3.5 | +29.5 | 1.3E-24 | <0.001 |
| GO:0009058 | biosynthetic process | 617 | 65.0 | 19.5 | +45.5 | 1.6E-21 | <0.001 |
| GO:0044249 | cellular biosynthetic process | 441 | 55.0 | 13.9 | +41.1 | 3.2E-21 | <0.001 |
| GO:0044260 | cellular macromolecule metabolic process | 381 | 50.0 | 12.1 | +37.9 | 4.6E-20 | <0.001 |
| GO:0005737 | cytoplasm | 656 | 63.0 | 20.7 | +42.3 | 1.5E-18 | <0.001 |
| GO:0044267 | cellular protein metabolic process | 279 | 40.0 | 8.8 | +31.2 | 3.4E-17 | <0.001 |
| GO:0044424 | intracellular part | 697 | 63.0 | 22.0 | +41.0 | 3.7E-17 | <0.001 |
| GO:0019538 | protein metabolic process | 285 | 40.0 | 9.0 | +31.0 | 7.3E-17 | <0.001 |
| GO:0005622 | intracellular | 835 | 64.0 | 26.4 | +37.6 | 8.5E-14 | <0.001 |
| GO:0033279 | ribosomal subunit | 13 | 9.0 | 0.4 | +8.6 | 1.6E-11 | <0.001 |
| GO:0044422 | organelle part | 28 | 11.0 | 0.9 | +10.1 | 2.8E-10 | <0.001 |
| GO:0044446 | intracellular organelle part | 28 | 11.0 | 0.9 | +10.1 | 2.8E-10 | <0.001 |
| GO:0000267 | cell fraction | 62 | 15.0 | 2.0 | +13.0 | 3.8E-10 | <0.001 |
| GO:0005624 | membrane fraction | 62 | 15.0 | 2.0 | +13.0 | 3.8E-10 | <0.001 |
| GO:0044238 | primary metabolic process | 1500 | 81.0 | 47.4 | +33.6 | 1.6E-9 | <0.001 |
| GO:0044237 | cellular metabolic process | 1627 | 85.0 | 51.5 | +33.5 | 2.0E-9 | <0.001 |
| GO:0043170 | macromolecule metabolic process | 1177 | 66.0 | 37.2 | +28.8 | 6.5E-8 | <0.001 |
| GO:0044464 | cell part | 2139 | 97.0 | 67.7 | +29.3 | 1.1E-7 | <0.001 |
| GO:0000049 | tRNA binding | 17 | 7.0 | 0.5 | +6.5 | 4.0E-7 | <0.001 |
| GO:0015934 | large ribosomal subunit | 7 | 5.0 | 0.2 | +4.8 | 5.9E-7 | <0.001 |
| GO:0003676 | nucleic acid binding | 588 | 40.0 | 18.6 | +21.4 | 8.5E-7 | <0.001 |
| GO:0008610 | lipid biosynthetic process | 126 | 16.0 | 4.0 | +12.0 | 1.5E-6 | <0.001 |
| GO:0044255 | cellular lipid metabolic process | 148 | 17.0 | 4.7 | +12.3 | 2.9E-6 | <0.001 |
| GO:0006629 | lipid metabolic process | 152 | 17.0 | 4.8 | +12.2 | 4.2E-6 | <0.001 |
| GO:0006633 | fatty acid biosynthetic process | 18 | 6.0 | 0.6 | +5.4 | 1.2E-5 | 0.002 |
| GO:0009103 | lipopolysaccharide biosynthetic process | 78 | 10.0 | 2.5 | +7.5 | 1.4E-4 | 0.020 |
| GO:0008653 | lipopolysaccharide metabolic process | 78 | 10.0 | 2.5 | +7.5 | 1.4E-4 | 0.020 |
| GO:0005515 | protein binding | 835 | 44.0 | 26.4 | +17.6 | 1.8E-4 | 0.026 |
| GO:0046394 | carboxylic acid biosynthetic process | 28 | 6.0 | 0.9 | +5.1 | 1.9E-4 | 0.026 |

## Fis – curated

| Category ID | Category Name | #Genes Category | #Genes Assigned | #Genes Expected | #Genes Enriched | p-value | Corrected p-value |
|---|---|---|---|---|---|---|---|
| GO:0048038 | quinone binding | 14 | 13.0 | 0.4 | +12.6 | 2.4E-19 | <0.001 |
| GO:0003954 | NADH dehydrogenase activity | 25 | 14.0 | 0.8 | +13.2 | 1.7E-15 | <0.001 |
| GO:0050136 | NADH dehydrogenase (quinone) activity | 24 | 13.0 | 0.8 | +12.2 | 3.3E-14 | <0.001 |
| GO:0016655 | oxidoreductase activity, acting on NADH or N... | 25 | 13.0 | 0.8 | +12.2 | 6.6E-14 | <0.001 |
| GO:0016651 | oxidoreductase activity, acting on NADH or N... | 33 | 14.0 | 1.0 | +13.0 | 2.5E-13 | <0.001 |
| GO:0042773 | ATP synthesis coupled electron transport | 21 | 10.0 | 0.7 | +9.3 | 1.9E-10 | <0.001 |
| GO:0008137 | NADH dehydrogenase (ubiquinone) activity | 21 | 10.0 | 0.7 | +9.3 | 1.9E-10 | <0.001 |
| GO:0006119 | oxidative phosphorylation | 31 | 10.0 | 1.0 | +9.0 | 1.8E-8 | <0.001 |
| GO:0009055 | electron carrier activity | 102 | 16.0 | 3.2 | +12.8 | 7.6E-8 | <0.001 |
| GO:0006120 | mitochondrial electron transport, NADH to ub... | 13 | 6.0 | 0.4 | +5.6 | 1.3E-6 | <0.001 |
| GO:0042775 | organelle ATP synthesis coupled electron tra... | 13 | 6.0 | 0.4 | +5.6 | 1.3E-6 | <0.001 |
| GO:0016310 | phosphorylation | 75 | 11.0 | 2.4 | +8.6 | 1.8E-5 | 0.002 |
| GO:0006796 | phosphate metabolic process | 80 | 11.0 | 2.5 | +8.5 | 3.4E-5 | 0.004 |
| GO:0006793 | phosphorus metabolic process | 81 | 11.0 | 2.6 | +8.4 | 3.8E-5 | 0.004 |
| GO:0006118 | electron transport | 236 | 19.0 | 7.5 | +11.5 | 1.3E-4 | 0.022 |
| GO:0048037 | cofactor binding | 201 | 17.0 | 6.4 | +10.6 | 1.6E-4 | 0.026 |
| GO:0016491 | oxidoreductase activity | 412 | 25.0 | 13.0 | +12.0 | 9.2E-4 | 0.114 |
| GO:0006091 | generation of precursor metabolites and ene... | 276 | 19.0 | 8.7 | +10.3 | 9.3E-4 | 0.114 |

## FNR – predicted

| Category ID | Category Name | #Genes Category | #Genes Assigned | #Genes Expected | #Genes Enriched | p-value | Corrected p-value |
|---|---|---|---|---|---|---|---|
| GO:0051539 | 4 iron, 4 sulfur cluster binding | 93 | 7.0 | 2.0 | +5.0 | 3.8E-3 | 0.262 |
| GO:0051540 | metal cluster binding | 126 | 8.0 | 2.7 | +5.3 | 5.8E-3 | 0.354 |
| GO:0051536 | iron-sulfur cluster binding | 126 | 8.0 | 2.7 | +5.3 | 5.8E-3 | 0.354 |
| GO:0009103 | lipopolysaccharide biosynthetic process | 83 | 6.0 | 1.8 | +4.2 | 8.9E-3 | 0.470 |
| GO:0008653 | lipopolysaccharide metabolic process | 83 | 6.0 | 1.8 | +4.2 | 8.9E-3 | 0.470 |

## FNR – curated

| Category ID | Category Name | #Genes Category | #Genes Assigned | #Genes Expected | #Genes Enriched | p-value | Corrected p-value |
|---|---|---|---|---|---|---|---|
| GO:0006118 | electron transport | 236 | 45.0 | 5.2 | +39.8 | 3.4E-33 | <0.001 |
| GO:0006091 | generation of precursor metabolites and ene... | 276 | 46.0 | 6.0 | +40.0 | 2.9E-31 | <0.001 |
| GO:0016491 | oxidoreductase activity | 412 | 46.0 | 9.0 | +37.0 | 2.4E-23 | <0.001 |
| GO:0009055 | electron carrier activity | 102 | 27.0 | 2.2 | +24.8 | 2.7E-23 | <0.001 |
| GO:0005506 | iron ion binding | 219 | 35.0 | 4.8 | +30.2 | 1.9E-22 | <0.001 |
| GO:0048038 | quinone binding | 14 | 13.0 | 0.3 | +12.7 | 1.5E-21 | <0.001 |
| GO:0046914 | transition metal ion binding | 458 | 43.0 | 10.0 | +33.0 | 1.4E-18 | <0.001 |
| GO:0003954 | NADH dehydrogenase activity | 25 | 14.0 | 0.5 | +13.5 | 7.6E-18 | <0.001 |
| GO:0043169 | cation binding | 511 | 43.0 | 11.2 | +31.8 | 9.7E-17 | <0.001 |
| GO:0050136 | NADH dehydrogenase (quinone) activity | 24 | 13.0 | 0.5 | +12.5 | 2.3E-16 | <0.001 |
| GO:0046872 | metal ion binding | 606 | 46.0 | 13.3 | +32.7 | 2.7E-16 | <0.001 |
| GO:0016655 | oxidoreductase activity, acting on NADH or N... | 25 | 13.0 | 0.5 | +12.5 | 4.6E-16 | <0.001 |
| GO:0043167 | ion binding | 622 | 46.0 | 13.6 | +32.4 | 7.6E-16 | <0.001 |
| GO:0016651 | oxidoreductase activity, acting on NADH or N... | 33 | 14.0 | 0.7 | +13.3 | 1.2E-15 | <0.001 |
| GO:0017004 | cytochrome complex assembly | 10 | 9.0 | 0.2 | +8.8 | 7.6E-15 | <0.001 |
| GO:0051540 | metal cluster binding | 126 | 22.0 | 2.8 | +19.2 | 7.8E-15 | <0.001 |
| GO:0051536 | iron-sulfur cluster binding | 126 | 22.0 | 2.8 | +19.2 | 7.8E-15 | <0.001 |
| GO:0006461 | protein complex assembly | 14 | 10.0 | 0.3 | +9.7 | 1.4E-14 | <0.001 |
| GO:0042126 | nitrate metabolic process | 19 | 11.0 | 0.4 | +10.6 | 2.0E-14 | <0.001 |
| GO:0042128 | nitrate assimilation | 19 | 11.0 | 0.4 | +10.6 | 2.0E-14 | <0.001 |
| GO:0051539 | 4 iron, 4 sulfur cluster binding | 93 | 19.0 | 2.0 | +17.0 | 3.4E-14 | <0.001 |
| GO:0043623 | cellular protein complex assembly | 11 | 9.0 | 0.2 | +8.8 | 4.1E-14 | <0.001 |
| GO:0042773 | ATP synthesis coupled electron transport | 21 | 10.0 | 0.5 | +9.5 | 4.4E-12 | <0.001 |
| GO:0008137 | NADH dehydrogenase (ubiquinone) activity | 21 | 10.0 | 0.5 | +9.5 | 4.4E-12 | <0.001 |
| GO:0016661 | oxidoreductase activity, acting on other nitrog... | 16 | 9.0 | 0.4 | +8.6 | 7.8E-12 | <0.001 |
| GO:0065003 | macromolecular complex assembly | 26 | 10.0 | 0.6 | +9.4 | 6.1E-11 | <0.001 |
| GO:0022607 | cellular component assembly | 27 | 10.0 | 0.6 | +9.4 | 9.5E-11 | <0.001 |
| GO:0006119 | oxidative phosphorylation | 31 | 10.0 | 0.7 | +9.3 | 4.7E-10 | <0.001 |
| GO:0015232 | heme transporter activity | 5 | 5.0 | 0.1 | +4.9 | 4.5E-9 | <0.001 |
| GO:0051184 | cofactor transporter activity | 6 | 5.0 | 0.1 | +4.9 | 2.7E-8 | <0.001 |
| GO:0006120 | mitochondrial electron transport, NADH to ub... | 13 | 6.0 | 0.3 | +5.7 | 1.4E-7 | <0.001 |
| GO:0042775 | organelle ATP synthesis coupled electron tra... | 13 | 6.0 | 0.3 | +5.7 | 1.4E-7 | <0.001 |
| GO:0048037 | cofactor binding | 201 | 18.0 | 4.4 | +13.6 | 2.0E-7 | <0.001 |
| GO:0016310 | phosphorylation | 75 | 11.0 | 1.6 | +9.4 | 4.7E-7 | <0.001 |
| GO:0008940 | nitrate reductase activity | 9 | 5.0 | 0.2 | +4.8 | 5.3E-7 | <0.001 |
| GO:0006810 | transport | 772 | 37.0 | 16.9 | +20.1 | 6.3E-7 | <0.001 |
| GO:0016151 | nickel ion binding | 25 | 7.0 | 0.5 | +6.5 | 6.7E-7 | <0.001 |
| GO:0051234 | establishment of localization | 782 | 37.0 | 17.1 | +19.9 | 8.7E-7 | <0.001 |
| GO:0006796 | phosphate metabolic process | 80 | 11.0 | 1.8 | +9.2 | 9.1E-7 | <0.001 |

## H-NS – predicted

| Category ID | Category Name | #Genes Category | #Genes Assigned | #Genes Expected | #Genes Enriched | p-value | Corrected p-value |
|---|---|---|---|---|---|---|---|
| GO:0032196 | transposition | 42 | 5.0 | 0.6 | +4.4 | 2.4E-4 | 0.024 |
| GO:0006313 | transposition, DNA-mediated | 42 | 5.0 | 0.6 | +4.4 | 2.4E-4 | 0.024 |
| GO:0043565 | sequence-specific DNA binding | 74 | 6.0 | 1.0 | +5.0 | 4.7E-4 | 0.042 |
| GO:0003677 | DNA binding | 443 | 15.0 | 6.1 | +8.9 | 7.1E-4 | 0.050 |
| GO:0006310 | DNA recombination | 66 | 5.0 | 0.9 | +4.1 | 2.0E-3 | 0.120 |
| GO:0003676 | nucleic acid binding | 588 | 16.0 | 8.1 | +7.9 | 4.7E-3 | 0.250 |

## H-NS – curated

| Category ID | Category Name | #Genes Category | #Genes Assigned | #Genes Expected | #Genes Enriched | p-value | Corrected p-value |
|---|---|---|---|---|---|---|---|
| GO:0015628 | protein secretion by the type II secretion syst... | 12 | 8.0 | 0.2 | +7.8 | 3.8E-13 | <0.001 |
| GO:0015627 | type II protein secretion system complex | 12 | 8.0 | 0.2 | +7.8 | 3.8E-13 | <0.001 |
| GO:0009306 | protein secretion | 40 | 9.0 | 0.6 | +8.4 | 1.9E-9 | <0.001 |
| GO:0008565 | protein transporter activity | 41 | 9.0 | 0.6 | +8.4 | 2.4E-9 | <0.001 |
| GO:0046903 | secretion | 42 | 9.0 | 0.6 | +8.4 | 3.0E-9 | <0.001 |
| GO:0032940 | secretion by cell | 42 | 9.0 | 0.6 | +8.4 | 3.0E-9 | <0.001 |
| GO:0051649 | establishment of cellular localization | 46 | 9.0 | 0.6 | +8.4 | 7.1E-9 | <0.001 |
| GO:0051641 | cellular localization | 47 | 9.0 | 0.6 | +8.4 | 8.6E-9 | <0.001 |
| GO:0009289 | fimbrium | 52 | 9.0 | 0.7 | +8.3 | 2.2E-8 | <0.001 |
| GO:0042995 | cell projection | 92 | 11.0 | 1.3 | +9.7 | 2.9E-8 | <0.001 |
| GO:0033036 | macromolecule localization | 79 | 10.0 | 1.1 | +8.9 | 7.7E-8 | <0.001 |
| GO:0008104 | protein localization | 79 | 10.0 | 1.1 | +8.9 | 7.7E-8 | <0.001 |
| GO:0045184 | establishment of protein localization | 79 | 10.0 | 1.1 | +8.9 | 7.7E-8 | <0.001 |
| GO:0043234 | protein complex | 110 | 9.0 | 1.5 | +7.5 | 1.5E-5 | <0.001 |
| GO:0016043 | cellular component organization and biogen... | 205 | 12.0 | 2.8 | +9.2 | 1.6E-5 | <0.001 |
| GO:0007155 | cell adhesion | 31 | 5.0 | 0.4 | +4.6 | 5.4E-5 | 0.002 |
| GO:0044464 | cell part | 2139 | 43.0 | 29.5 | +13.5 | 2.3E-4 | 0.016 |
| GO:0006810 | transport | 772 | 22.0 | 10.6 | +11.4 | 3.3E-4 | 0.026 |
| GO:0051234 | establishment of localization | 782 | 22.0 | 10.8 | +11.2 | 4.0E-4 | 0.034 |
| GO:0022892 | substrate-specific transporter activity | 324 | 12.0 | 4.5 | +7.5 | 1.3E-3 | 0.096 |

IHF - predicted

| Category ID | Category Name | #Genes Category | #Genes Assigned | #Genes Expected | #Genes Enriched | p-value | Corrected p-value |
|---|---|---|---|---|---|---|---|
| GO:0009103 | lipopolysaccharide biosynthetic process | 78 | 19.0 | 3.0 | +16.0 | 4.0E-11 | <0.001 |
| GO:0008653 | lipopolysaccharide metabolic process | 78 | 19.0 | 3.0 | +16.0 | 4.0E-11 | <0.001 |
| GO:0000271 | polysaccharide biosynthetic process | 99 | 19.0 | 3.8 | +15.2 | 3.2E-9 | <0.001 |
| GO:0005976 | polysaccharide metabolic process | 105 | 19.0 | 4.0 | +15.0 | 8.9E-9 | <0.001 |
| GO:0044264 | cellular polysaccharide metabolic process | 105 | 19.0 | 4.0 | +15.0 | 8.9E-9 | <0.001 |
| GO:0043284 | biopolymer biosynthetic process | 108 | 19.0 | 4.1 | +14.9 | 1.4E-8 | <0.001 |
| GO:0044255 | cellular lipid metabolic process | 148 | 22.0 | 5.7 | +16.3 | 2.5E-8 | <0.001 |
| GO:0006091 | generation of precursor metabolites and ene... | 276 | 31.0 | 10.6 | +20.4 | 2.7E-8 | <0.001 |
| GO:0006629 | lipid metabolic process | 152 | 22.0 | 5.8 | +16.2 | 4.1E-8 | <0.001 |
| GO:0006536 | glutamate metabolic process | 12 | 7.0 | 0.5 | +6.5 | 7.1E-8 | <0.001 |
| GO:0009060 | aerobic respiration | 23 | 9.0 | 0.9 | +8.1 | 7.3E-8 | <0.001 |
| GO:0019544 | arginine catabolic process to glutamate | 5 | 5.0 | 0.2 | +4.8 | 7.7E-8 | <0.001 |
| GO:0045333 | cellular respiration | 30 | 10.0 | 1.1 | +8.9 | 8.0E-8 | <0.001 |
| GO:0044262 | cellular carbohydrate metabolic process | 249 | 28.0 | 9.5 | +18.5 | 1.4E-7 | <0.001 |
| GO:0006118 | electron transport | 236 | 27.0 | 9.0 | +18.0 | 1.7E-7 | <0.001 |
| GO:0008610 | lipid biosynthetic process | 126 | 19.0 | 4.8 | +14.2 | 1.9E-7 | <0.001 |
| GO:0006527 | arginine catabolic process | 6 | 5.0 | 0.2 | +4.8 | 4.5E-7 | <0.001 |
| GO:0009109 | coenzyme catabolic process | 22 | 8.0 | 0.8 | +7.2 | 7.9E-7 | <0.001 |
| GO:0051187 | cofactor catabolic process | 22 | 8.0 | 0.8 | +7.2 | 7.9E-7 | <0.001 |
| GO:0006099 | tricarboxylic acid cycle | 22 | 8.0 | 0.8 | +7.2 | 7.9E-7 | <0.001 |
| GO:0046356 | acetyl-CoA catabolic process | 22 | 8.0 | 0.8 | +7.2 | 7.9E-7 | <0.001 |
| GO:0016051 | carbohydrate biosynthetic process | 140 | 19.0 | 5.4 | +13.6 | 1.0E-6 | <0.001 |
| GO:0009065 | glutamine family amino acid catabolic proce... | 7 | 5.0 | 0.3 | +4.7 | 1.5E-6 | <0.001 |
| GO:0009288 | flagellin-based flagellum | 24 | 8.0 | 0.9 | +7.1 | 1.7E-6 | <0.001 |
| GO:0006084 | acetyl-CoA metabolic process | 25 | 8.0 | 1.0 | +7.0 | 2.4E-6 | <0.001 |
| GO:0015980 | energy derivation by oxidation of organic com... | 43 | 10.0 | 1.6 | +8.4 | 3.3E-6 | <0.001 |
| GO:0016491 | oxidoreductase activity | 412 | 34.0 | 15.8 | +18.2 | 9.0E-6 | 0.002 |
| GO:0009063 | amino acid catabolic process | 15 | 6.0 | 0.6 | +5.4 | 1.1E-5 | 0.002 |
| GO:0019861 | flagellum | 40 | 9.0 | 1.5 | +7.5 | 1.4E-5 | 0.002 |
| GO:0009310 | amine catabolic process | 17 | 6.0 | 0.7 | +5.3 | 2.5E-5 | 0.004 |
| GO:0044270 | nitrogen compound catabolic process | 17 | 6.0 | 0.7 | +5.3 | 2.5E-5 | 0.004 |

IHF – curated

| Category ID | Category Name | #Genes Category | #Genes Assigned | #Genes Expected | #Genes Enriched | p-value | Corrected p-value |
|---|---|---|---|---|---|---|---|
| GO:0003954 | NADH dehydrogenase activity | 25 | 17.0 | 1.0 | +16.0 | 2.9E-19 | <0.001 |
| GO:0048038 | quinone binding | 14 | 13.0 | 0.5 | +12.5 | 3.2E-18 | <0.001 |
| GO:0050136 | NADH dehydrogenase (quinone) activity | 24 | 16.0 | 0.9 | +15.1 | 5.7E-18 | <0.001 |
| GO:0016655 | oxidoreductase activity, acting on NADH or N... | 25 | 16.0 | 1.0 | +15.0 | 1.5E-17 | <0.001 |
| GO:0006091 | generation of precursor metabolites and ene... | 276 | 43.0 | 10.6 | +32.4 | 1.7E-16 | <0.001 |
| GO:0016651 | oxidoreductase activity, acting on NADH or N... | 33 | 17.0 | 1.3 | +15.7 | 2.4E-16 | <0.001 |
| GO:0009055 | electron carrier activity | 102 | 26.0 | 3.9 | +22.1 | 2.1E-15 | <0.001 |
| GO:0016491 | oxidoreductase activity | 412 | 51.0 | 15.8 | +35.2 | 2.9E-15 | <0.001 |
| GO:0006118 | electron transport | 236 | 38.0 | 9.0 | +29.0 | 4.8E-15 | <0.001 |
| GO:0042773 | ATP synthesis coupled electron transport | 21 | 13.0 | 0.8 | +12.2 | 3.7E-14 | <0.001 |
| GO:0008137 | NADH dehydrogenase (ubiquinone) activity | 21 | 13.0 | 0.8 | +12.2 | 3.7E-14 | <0.001 |
| GO:0006119 | oxidative phosphorylation | 31 | 13.0 | 1.2 | +11.8 | 2.7E-11 | <0.001 |
| GO:0048037 | cofactor binding | 201 | 28.0 | 7.7 | +20.3 | 1.1E-9 | <0.001 |
| GO:0042775 | organelle ATP synthesis coupled electron tra... | 13 | 8.0 | 0.5 | +7.5 | 4.3E-9 | <0.001 |
| GO:0006120 | mitochondrial electron transport, NADH to ub... | 13 | 8.0 | 0.5 | +7.5 | 4.3E-9 | <0.001 |
| GO:0051540 | metal cluster binding | 126 | 20.0 | 4.8 | +15.2 | 3.7E-8 | <0.001 |
| GO:0051536 | iron-sulfur cluster binding | 126 | 20.0 | 4.8 | +15.2 | 3.7E-8 | <0.001 |
| GO:0051539 | 4 iron, 4 sulfur cluster binding | 93 | 17.0 | 3.6 | +13.4 | 4.9E-8 | <0.001 |
| GO:0016310 | phosphorylation | 75 | 15.0 | 2.9 | +12.1 | 9.0E-8 | <0.001 |
| GO:0005506 | iron ion binding | 219 | 26.0 | 8.4 | +17.6 | 1.4E-7 | <0.001 |
| GO:0042126 | nitrate metabolic process | 19 | 8.0 | 0.7 | +7.3 | 2.1E-7 | <0.001 |
| GO:0042128 | nitrate assimilation | 19 | 8.0 | 0.7 | +7.3 | 2.1E-7 | <0.001 |
| GO:0006796 | phosphate metabolic process | 80 | 15.0 | 3.1 | +11.9 | 2.2E-7 | <0.001 |
| GO:0006793 | phosphorus metabolic process | 81 | 15.0 | 3.1 | +11.9 | 2.6E-7 | <0.001 |
| GO:0016661 | oxidoreductase activity, acting on other nitrog... | 16 | 7.0 | 0.6 | +6.4 | 9.1E-7 | <0.001 |
| GO:0006082 | organic acid metabolic process | 312 | 28.0 | 11.9 | +16.1 | 1.4E-5 | 0.004 |
| GO:0046914 | transition metal ion binding | 458 | 36.0 | 17.5 | +18.5 | 1.4E-5 | 0.004 |
| GO:0006817 | phosphate transport | 10 | 5.0 | 0.4 | +4.6 | 1.7E-5 | 0.004 |
| GO:0046872 | metal ion binding | 606 | 43.0 | 23.2 | +19.8 | 2.3E-5 | 0.004 |
| GO:0019752 | carboxylic acid metabolic process | 307 | 27.0 | 11.8 | +15.2 | 2.9E-5 | 0.006 |
| GO:0043167 | ion binding | 622 | 43.0 | 23.8 | +19.2 | 4.4E-5 | 0.008 |
| GO:0006807 | nitrogen compound metabolic process | 266 | 24.0 | 10.2 | +13.8 | 5.6E-5 | 0.010 |
| GO:0043169 | cation binding | 511 | 37.0 | 19.6 | +17.4 | 6.6E-5 | 0.012 |
| GO:0009082 | branched chain family amino acid biosynthet... | 17 | 5.0 | 0.7 | +4.3 | 3.3E-4 | 0.038 |
| GO:0015198 | oligopeptide transporter activity | 17 | 5.0 | 0.7 | +4.3 | 3.3E-4 | 0.038 |
| GO:0006810 | transport | 772 | 47.0 | 29.6 | +17.4 | 4.2E-4 | 0.052 |
| GO:0050662 | coenzyme binding | 132 | 14.0 | 5.1 | +8.9 | 4.4E-4 | 0.060 |
| GO:0015698 | inorganic anion transport | 28 | 6.0 | 1.1 | +4.9 | 5.4E-4 | 0.066 |
| GO:0051234 | establishment of localization | 782 | 47.0 | 29.9 | +17.1 | 5.7E-4 | 0.068 |

## NarL - predicted

| Category ID | Category Name | #Genes Category | #Genes Assigned | #Genes Expected | #Genes Enriched | p-value | Corrected p-value |
|---|---|---|---|---|---|---|---|
| GO:0016151 | nickel ion binding | 25 | 6.0 | 0.3 | +5.7 | 3.1E-7 | <0.001 |
| GO:0006812 | cation transport | 113 | 9.0 | 1.3 | +7.7 | 5.3E-6 | <0.001 |
| GO:0042625 | ATPase activity, coupled to transmembrane ... | 28 | 5.0 | 0.3 | +4.7 | 1.6E-5 | 0.002 |
| GO:0046872 | metal ion binding | 606 | 19.0 | 7.2 | +11.8 | 3.0E-5 | 0.006 |
| GO:0043167 | ion binding | 622 | 19.0 | 7.4 | +11.6 | 4.3E-5 | 0.006 |
| GO:0042626 | ATPase activity, coupled to transmembrane ... | 58 | 6.0 | 0.7 | +5.3 | 5.3E-5 | 0.006 |
| GO:0043492 | ATPase activity, coupled to movement of sub... | 58 | 6.0 | 0.7 | +5.3 | 5.3E-5 | 0.006 |
| GO:0016820 | hydrolase activity, acting on acid anhydrides, ... | 60 | 6.0 | 0.7 | +5.3 | 6.4E-5 | 0.006 |
| GO:0015399 | primary active transmembrane transporter a... | 64 | 6.0 | 0.8 | +5.2 | 9.2E-5 | 0.008 |
| GO:0015405 | P-P-bond-hydrolysis-driven transmembrane ... | 64 | 6.0 | 0.8 | +5.2 | 9.2E-5 | 0.008 |
| GO:0030001 | metal ion transport | 97 | 7.0 | 1.2 | +5.8 | 1.2E-4 | 0.010 |
| GO:0006811 | ion transport | 171 | 9.0 | 2.0 | +7.0 | 1.5E-4 | 0.012 |
| GO:0043169 | cation binding | 511 | 16.0 | 6.1 | +9.9 | 1.7E-4 | 0.016 |
| GO:0022890 | inorganic cation transmembrane transporter... | 48 | 5.0 | 0.6 | +4.4 | 2.3E-4 | 0.016 |
| GO:0042623 | ATPase activity, coupled | 79 | 6.0 | 0.9 | +5.1 | 3.0E-4 | 0.020 |
| GO:0000041 | transition metal ion transport | 56 | 5.0 | 0.7 | +4.3 | 4.7E-4 | 0.030 |
| GO:0046914 | transition metal ion binding | 458 | 14.0 | 5.4 | +8.6 | 6.3E-4 | 0.040 |
| GO:0051540 | metal cluster binding | 126 | 7.0 | 1.5 | +5.5 | 6.3E-4 | 0.042 |
| GO:0051536 | iron-sulfur cluster binding | 126 | 7.0 | 1.5 | +5.5 | 6.3E-4 | 0.042 |
| GO:0051539 | 4 iron, 4 sulfur cluster binding | 93 | 6.0 | 1.1 | +4.9 | 7.2E-4 | 0.054 |
| GO:0022891 | substrate-specific transmembrane transport... | 266 | 10.0 | 3.2 | +6.8 | 9.3E-4 | 0.064 |
| GO:0006091 | generation of precursor metabolites and ene... | 276 | 10.0 | 3.3 | +6.7 | 1.2E-3 | 0.080 |
| GO:0043565 | sequence-specific DNA binding | 74 | 5.0 | 0.9 | +4.1 | 1.7E-3 | 0.104 |
| GO:0016310 | phosphorylation | 75 | 5.0 | 0.9 | +4.1 | 1.8E-3 | 0.112 |
| GO:0015075 | ion transmembrane transporter activity | 111 | 6.0 | 1.3 | +4.7 | 1.8E-3 | 0.112 |
| GO:0005524 | ATP binding | 347 | 11.0 | 4.1 | +6.9 | 2.0E-3 | 0.120 |
| GO:0000160 | two-component signal transduction system (... | 78 | 5.0 | 0.9 | +4.1 | 2.1E-3 | 0.128 |
| GO:0032559 | adenyl ribonucleotide binding | 349 | 11.0 | 4.1 | +6.9 | 2.1E-3 | 0.130 |
| GO:0008324 | cation transmembrane transporter activity | 79 | 5.0 | 0.9 | +4.1 | 2.3E-3 | 0.134 |
| GO:0006796 | phosphate metabolic process | 80 | 5.0 | 1.0 | +4.0 | 2.4E-3 | 0.146 |
| GO:0006793 | phosphorus metabolic process | 81 | 5.0 | 1.0 | +4.0 | 2.5E-3 | 0.152 |
| GO:0022857 | transmembrane transporter activity | 310 | 10.0 | 3.7 | +6.3 | 3.0E-3 | 0.160 |
| GO:0022804 | active transmembrane transporter activity | 220 | 8.0 | 2.6 | +5.4 | 3.9E-3 | 0.192 |
| GO:0022892 | substrate-specific transporter activity | 324 | 10.0 | 3.9 | +6.1 | 4.1E-3 | 0.194 |
| GO:0032555 | purine ribonucleotide binding | 380 | 11.0 | 4.5 | +6.5 | 4.2E-3 | 0.198 |
| GO:0032553 | ribonucleotide binding | 380 | 11.0 | 4.5 | +6.5 | 4.2E-3 | 0.198 |
| GO:0004871 | signal transducer activity | 92 | 5.0 | 1.1 | +3.9 | 4.4E-3 | 0.216 |
| GO:0016887 | ATPase activity | 133 | 6.0 | 1.6 | +4.4 | 4.5E-3 | 0.218 |
| GO:0030554 | adenyl nucleotide binding | 390 | 11.0 | 4.6 | +6.4 | 5.1E-3 | 0.228 |

## NarL - curated

| Category ID | Category Name | #Genes Category | #Genes Assigned | #Genes Expected | #Genes Enriched | p-value | Corrected p-value |
|---|---|---|---|---|---|---|---|
| GO:0006118 | electron transport | 236 | 27.0 | 2.8 | +24.2 | 1.4E-21 | <0.001 |
| GO:0005506 | iron ion binding | 219 | 25.0 | 2.6 | +22.4 | 8.6E-20 | <0.001 |
| GO:0006091 | generation of precursor metabolites and ene... | 276 | 27.0 | 3.3 | +23.7 | 9.3E-20 | <0.001 |
| GO:0017004 | cytochrome complex assembly | 10 | 9.0 | 0.1 | +8.9 | 2.2E-17 | <0.001 |
| GO:0043623 | cellular protein complex assembly | 11 | 9.0 | 0.1 | +8.9 | 1.2E-16 | <0.001 |
| GO:0006461 | protein complex assembly | 14 | 9.0 | 0.2 | +8.8 | 4.3E-15 | <0.001 |
| GO:0016661 | oxidoreductase activity, acting on other nitrog... | 16 | 9.0 | 0.2 | +8.8 | 2.4E-14 | <0.001 |
| GO:0042126 | nitrate metabolic process | 19 | 9.0 | 0.2 | +8.8 | 1.9E-13 | <0.001 |
| GO:0042128 | nitrate assimilation | 19 | 9.0 | 0.2 | +8.8 | 1.9E-13 | <0.001 |
| GO:0051540 | metal cluster binding | 126 | 16.0 | 1.5 | +14.5 | 3.4E-13 | <0.001 |
| GO:0051536 | iron-sulfur cluster binding | 126 | 16.0 | 1.5 | +14.5 | 3.4E-13 | <0.001 |
| GO:0016491 | oxidoreductase activity | 412 | 25.0 | 4.9 | +20.1 | 3.8E-13 | <0.001 |
| GO:0051539 | 4 iron, 4 sulfur cluster binding | 93 | 14.0 | 1.1 | +12.9 | 1.2E-12 | <0.001 |
| GO:0046914 | transition metal ion binding | 458 | 25.0 | 5.4 | +19.6 | 4.3E-12 | <0.001 |
| GO:0065003 | macromolecular complex assembly | 26 | 9.0 | 0.3 | +8.7 | 6.0E-12 | <0.001 |
| GO:0022607 | cellular component assembly | 27 | 9.0 | 0.3 | +8.7 | 8.9E-12 | <0.001 |
| GO:0043169 | cation binding | 511 | 25.0 | 6.1 | +18.9 | 5.0E-11 | <0.001 |
| GO:0015232 | heme transporter activity | 5 | 5.0 | 0.1 | +4.9 | 1.9E-10 | <0.001 |
| GO:0051184 | cofactor transporter activity | 6 | 5.0 | 0.1 | +4.9 | 1.2E-9 | <0.001 |
| GO:0046872 | metal ion binding | 606 | 25.0 | 7.2 | +17.8 | 2.1E-9 | <0.001 |
| GO:0006810 | transport | 772 | 28.0 | 9.2 | +18.8 | 2.3E-9 | <0.001 |
| GO:0051234 | establishment of localization | 782 | 28.0 | 9.3 | +18.7 | 3.1E-9 | <0.001 |
| GO:0043167 | ion binding | 622 | 25.0 | 7.4 | +17.6 | 3.7E-9 | <0.001 |
| GO:0009055 | electron carrier activity | 102 | 11.0 | 1.2 | +9.8 | 1.7E-8 | <0.001 |
| GO:0008940 | nitrate reductase activity | 9 | 5.0 | 0.1 | +4.9 | 2.4E-8 | <0.001 |
| GO:0030151 | molybdenum ion binding | 23 | 5.0 | 0.3 | +4.7 | 5.6E-6 | <0.001 |
| GO:0042597 | periplasmic space | 168 | 10.0 | 2.0 | +8.0 | 2.0E-5 | 0.002 |
| GO:0045333 | cellular respiration | 30 | 5.0 | 0.4 | +4.6 | 2.2E-5 | 0.002 |
| GO:0044464 | cell part | 2139 | 39.0 | 25.4 | +13.6 | 6.8E-5 | 0.002 |
| GO:0015980 | energy derivation by oxidation of organic com... | 43 | 5.0 | 0.5 | +4.5 | 1.3E-4 | 0.006 |
| GO:0016020 | membrane | 1134 | 26.0 | 13.5 | +12.5 | 1.4E-4 | 0.006 |
| GO:0016021 | integral to membrane | 882 | 22.0 | 10.5 | +11.5 | 1.9E-4 | 0.008 |
| GO:0031224 | intrinsic to membrane | 882 | 22.0 | 10.5 | +11.5 | 1.9E-4 | 0.008 |
| GO:0006807 | nitrogen compound metabolic process | 266 | 11.0 | 3.2 | +7.8 | 2.2E-4 | 0.008 |
| GO:0044425 | membrane part | 945 | 22.0 | 11.2 | +10.8 | 5.4E-4 | 0.030 |
| GO:0016043 | cellular component organization and biogen... | 205 | 9.0 | 2.4 | +6.6 | 5.7E-4 | 0.030 |
| GO:0044267 | cellular protein metabolic process | 279 | 10.0 | 3.3 | +6.7 | 1.3E-3 | 0.064 |
| GO:0019538 | protein metabolic process | 285 | 10.0 | 3.4 | +6.6 | 1.6E-3 | 0.074 |
| GO:0043234 | protein complex | 110 | 6.0 | 1.3 | +4.7 | 1.7E-3 | 0.090 |

## 2. GO Analysis Results of Splits in DREM Map

In this section we present significant GO enrichments for the sets of genes assigned to the same path out of splits conditional on the set of genes going into the split. The split number and the path (i.e. whether it was the low, middle, or high path) is indicated above each table and corresponds to Figure 5A in the main manuscript.

Conditional on the set of genes passing the filtering criteria, the upregulated genes at the first split were most enriched for carbohydrate transport (p-val $<10^{-8}$), while the downregulated genes were most enriched for biosynthetic process genes (p-val $<10^{-30}$) including translation genes (p-val $<10^{-24}$). The most enriched biological process for the highest activated path conditioned on the set of genes going into split 2 was oxidoreductase activity (p-val $<8\times10^{-6}$). The lower path out of split 6 was enriched for genes related to the ribosome (p-val$< 10^{-11}$) and aerobic respiration (p-val $<4\times10^{-7}$). Genes on the lower path out of split 9, were enriched for genes known to be involved in aerobic and cellular respiration (p-val $< 2\times10^{-5}$), TCA cycle (p-val $< 4\times10^{-5}$), and coenzyme catabolic processes (p-val $< 4\times10^{-5}$). Additional GO category enrichments can be found in the tables below. Overall the GO categories found here among activated and repressed genes are consistent with the previously known literature on *E. coli* anaerobic response [1,2,3,4].

# Split 1 - Low

| Category ID | Category Name | #Genes Split in | #Genes on Path | #Genes Expected | #Genes Enriched | p-value | Corrected p-value |
|---|---|---|---|---|---|---|---|
| GO:0009058 | biosynthetic process | 358 | 246.0 | 146.8 | +99.2 | 8.8E-31 | <0.001 |
| GO:0044249 | cellular biosynthetic process | 259 | 189.0 | 106.2 | +82.8 | 1.9E-28 | <0.001 |
| GO:0044424 | intracellular part | 430 | 276.0 | 176.3 | +99.7 | 4.8E-27 | <0.001 |
| GO:0006412 | translation | 94 | 86.0 | 38.5 | +47.5 | 1.1E-25 | <0.001 |
| GO:0005737 | cytoplasm | 410 | 260.0 | 168.1 | +91.9 | 4.9E-24 | <0.001 |
| GO:0009059 | macromolecule biosynthetic process | 179 | 137.0 | 73.4 | +63.6 | 8.5E-24 | <0.001 |
| GO:0005622 | intracellular | 501 | 302.0 | 205.4 | +96.6 | 6.1E-23 | <0.001 |
| GO:0043228 | non-membrane-bound organelle | 69 | 66.0 | 28.3 | +37.7 | 8.5E-23 | <0.001 |
| GO:0043232 | intracellular non-membrane-bound organelle | 69 | 66.0 | 28.3 | +37.7 | 8.5E-23 | <0.001 |
| GO:0043229 | intracellular organelle | 76 | 71.0 | 31.2 | +39.8 | 1.0E-22 | <0.001 |
| GO:0030529 | ribonucleoprotein complex | 57 | 56.0 | 23.4 | +32.6 | 2.7E-21 | <0.001 |
| GO:0044237 | cellular metabolic process | 922 | 487.0 | 378.0 | +109.0 | 4.3E-21 | <0.001 |
| GO:0005840 | ribosome | 56 | 55.0 | 23.0 | +32.0 | 6.7E-21 | <0.001 |
| GO:0003735 | structural constituent of ribosome | 55 | 54.0 | 22.6 | +31.4 | 1.7E-20 | <0.001 |
| GO:0044238 | primary metabolic process | 862 | 459.0 | 353.4 | +105.6 | 2.5E-20 | <0.001 |
| GO:0003723 | RNA binding | 82 | 72.0 | 33.6 | +38.4 | 4.1E-19 | <0.001 |
| GO:0044444 | cytoplasmic part | 74 | 65.0 | 30.3 | +34.7 | 2.4E-17 | <0.001 |
| GO:0044260 | cellular macromolecule metabolic process | 265 | 170.0 | 108.7 | +61.3 | 5.8E-16 | <0.001 |
| GO:0019843 | rRNA binding | 36 | 36.0 | 14.8 | +21.2 | 7.7E-15 | <0.001 |
| GO:0044267 | cellular protein metabolic process | 200 | 132.0 | 82.0 | +50.0 | 7.7E-14 | <0.001 |
| GO:0043170 | macromolecule metabolic process | 683 | 360.0 | 280.0 | +80.0 | 1.2E-13 | <0.001 |
| GO:0019538 | protein metabolic process | 204 | 132.0 | 83.6 | +48.4 | 7.6E-13 | <0.001 |
| GO:0003676 | nucleic acid binding | 311 | 179.0 | 127.5 | +51.5 | 1.9E-10 | <0.001 |
| GO:0006399 | tRNA metabolic process | 39 | 35.0 | 16.0 | +19.0 | 2.2E-10 | <0.001 |
| GO:0006396 | RNA processing | 39 | 34.0 | 16.0 | +18.0 | 2.3E-9 | <0.001 |
| GO:0044464 | cell part | 1241 | 578.0 | 508.8 | +69.2 | 2.8E-9 | <0.001 |
| GO:0006139 | nucleobase, nucleoside, nucleotide and nuc... | 377 | 204.0 | 154.6 | +49.4 | 1.4E-8 | <0.001 |
| GO:0008610 | lipid biosynthetic process | 82 | 58.0 | 33.6 | +24.4 | 2.9E-8 | <0.001 |
| GO:0044422 | organelle part | 19 | 19.0 | 7.8 | +11.2 | 4.0E-8 | <0.001 |
| GO:0044446 | intracellular organelle part | 19 | 19.0 | 7.8 | +11.2 | 4.0E-8 | <0.001 |
| GO:0043283 | biopolymer metabolic process | 398 | 210.0 | 163.2 | +46.8 | 1.3E-7 | <0.001 |
| GO:0006629 | lipid metabolic process | 94 | 62.0 | 38.5 | +23.5 | 5.5E-7 | <0.001 |
| GO:0044255 | cellular lipid metabolic process | 93 | 61.0 | 38.1 | +22.9 | 9.2E-7 | <0.001 |
| GO:0016874 | ligase activity | 53 | 39.0 | 21.7 | +17.3 | 1.2E-6 | <0.001 |
| GO:0005515 | protein binding | 471 | 237.0 | 193.1 | +43.9 | 3.0E-6 | 0.002 |
| GO:0006732 | coenzyme metabolic process | 62 | 43.0 | 25.4 | +17.6 | 4.5E-6 | 0.004 |

## Split 1 - High

| Category ID | Category Name | #Genes Split in | #Genes on Path | #Genes Expected | #Genes Enriched | p-value | Corrected p-value |
|---|---|---|---|---|---|---|---|
| GO:0008643 | carbohydrate transport | 79 | 70.0 | 46.6 | +23.4 | 4.5E-9 | <0.001 |
| GO:0015144 | carbohydrate transmembrane transporter ac... | 77 | 68.0 | 45.4 | +22.6 | 1.1E-8 | <0.001 |
| GO:0051119 | sugar transmembrane transporter activity | 77 | 68.0 | 45.4 | +22.6 | 1.1E-8 | <0.001 |
| GO:0005351 | sugar:hydrogen ion symporter activity | 77 | 68.0 | 45.4 | +22.6 | 1.1E-8 | <0.001 |
| GO:0009401 | phosphoenolpyruvate-dependent sugar pho... | 42 | 40.0 | 24.8 | +15.2 | 8.6E-8 | <0.001 |
| GO:0015293 | symporter activity | 91 | 76.0 | 53.7 | +22.3 | 2.8E-7 | <0.001 |
| GO:0007165 | signal transduction | 88 | 73.0 | 51.9 | +21.1 | 8.5E-7 | <0.001 |
| GO:0015291 | secondary active transmembrane transporte... | 109 | 86.0 | 64.3 | +21.7 | 5.7E-6 | <0.001 |
| GO:0007154 | cell communication | 95 | 76.0 | 56.0 | +20.0 | 8.0E-6 | <0.001 |
| GO:0008982 | protein-N(PI)-phosphohistidine-sugar phosp... | 22 | 22.0 | 13.0 | +9.0 | 8.5E-6 | 0.002 |
| GO:0006810 | transport | 469 | 317.0 | 276.7 | +40.3 | 1.2E-5 | 0.002 |
| GO:0031224 | intrinsic to membrane | 487 | 327.0 | 287.3 | +39.7 | 2.1E-5 | 0.006 |
| GO:0016021 | integral to membrane | 487 | 327.0 | 287.3 | +39.7 | 2.1E-5 | 0.006 |
| GO:0051234 | establishment of localization | 471 | 317.0 | 277.9 | +39.1 | 2.2E-5 | 0.008 |
| GO:0044425 | membrane part | 531 | 349.0 | 313.3 | +35.7 | 1.8E-4 | 0.036 |

## Split 2 - Low

| Category ID | Category Name | #Genes Split in | #Genes on Path | #Genes Expected | #Genes Enriched | p-value | Corrected p-value |
|---|---|---|---|---|---|---|---|
| GO:0009289 | fimbrium | 23 | 18.0 | 8.3 | +9.7 | 4.1E-5 | 0.002 |

## Split 2 - Middle

| Category ID | Category Name | #Genes Split in | #Genes on Path | #Genes Expected | #Genes Enriched | p-value | Corrected p-value |
|---|---|---|---|---|---|---|---|
| GO:0043167 | ion binding | 226 | 154.0 | 131.1 | +22.9 | 4.2E-4 | 0.020 |
| GO:0006811 | ion transport | 54 | 43.0 | 31.3 | +11.7 | 5.6E-4 | 0.030 |
| GO:0046872 | metal ion binding | 216 | 147.0 | 125.3 | +21.7 | 6.4E-4 | 0.036 |

## Split 2 - High

| Category ID | Category Name | #Genes Split in | #Genes on Path | #Genes Expected | #Genes Enriched | p-value | Corrected p-value |
|---|---|---|---|---|---|---|---|
| GO:0016491 | oxidoreductase activity | 156 | 23.0 | 9.0 | +14.0 | 7.7E-6 | <0.001 |
| GO:0042126 | nitrate metabolic process | 12 | 6.0 | 0.7 | +5.3 | 2.2E-5 | 0.002 |
| GO:0042128 | nitrate assimilation | 12 | 6.0 | 0.7 | +5.3 | 2.2E-5 | 0.002 |
| GO:0006091 | generation of precursor metabolites and ene... | 111 | 17.0 | 6.4 | +10.6 | 9.8E-5 | 0.008 |
| GO:0006950 | response to stress | 44 | 10.0 | 2.5 | +7.5 | 1.2E-4 | 0.008 |
| GO:0006118 | electron transport | 102 | 16.0 | 5.9 | +10.1 | 1.2E-4 | 0.010 |
| GO:0016661 | oxidoreductase activity, acting on other nitrog... | 13 | 5.0 | 0.8 | +4.2 | 5.1E-4 | 0.032 |
| GO:0016614 | oxidoreductase activity, acting on CH-OH gro... | 21 | 6.0 | 1.2 | +4.8 | 8.3E-4 | 0.046 |

## Split 3 - High

| Category ID | Category Name | #Genes Split in | #Genes on Path | #Genes Expected | #Genes Enriched | p-value | Corrected p-value |
|---|---|---|---|---|---|---|---|
| GO:0006091 | generation of precursor metabolites and ene... | 73 | 47.0 | 30.3 | +16.7 | 3.0E-5 | 0.002 |
| GO:0051540 | metal cluster binding | 35 | 26.0 | 14.5 | +11.5 | 6.0E-5 | 0.004 |
| GO:0051536 | iron-sulfur cluster binding | 35 | 26.0 | 14.5 | +11.5 | 6.0E-5 | 0.004 |
| GO:0006118 | electron transport | 67 | 43.0 | 27.8 | +15.2 | 7.8E-5 | 0.004 |
| GO:0051539 | 4 iron, 4 sulfur cluster binding | 27 | 21.0 | 11.2 | +9.8 | 1.1E-4 | 0.006 |

## Split 5- High

| Category ID | Category Name | #Genes Split in | #Genes on Path | #Genes Expected | #Genes Enriched | p-value | Corrected p-value |
|---|---|---|---|---|---|---|---|
| GO:0016020 | membrane | 142 | 116.0 | 99.5 | +16.5 | 1.9E-4 | 0.002 |

# Split 6 - Low

| Category ID | Category Name | #Genes Split in | #Genes on Path | #Genes Expected | #Genes Enriched | p-value | Corrected p-value |
|---|---|---|---|---|---|---|---|
| GO:0044444 | cytoplasmic part | 65 | 37.0 | 12.5 | +24.5 | 1.9E-12 | <0.001 |
| GO:0019843 | rRNA binding | 36 | 26.0 | 6.9 | +19.1 | 2.0E-12 | <0.001 |
| GO:0003735 | structural constituent of ribosome | 54 | 33.0 | 10.3 | +22.7 | 2.3E-12 | <0.001 |
| GO:0005840 | ribosome | 55 | 33.0 | 10.5 | +22.5 | 4.8E-12 | <0.001 |
| GO:0030529 | ribonucleoprotein complex | 56 | 33.0 | 10.7 | +22.3 | 9.7E-12 | <0.001 |
| GO:0043229 | intracellular organelle | 71 | 37.0 | 13.6 | +23.4 | 7.0E-11 | <0.001 |
| GO:0044424 | intracellular part | 276 | 88.0 | 52.9 | +35.1 | 5.8E-10 | <0.001 |
| GO:0043228 | non-membrane-bound organelle | 66 | 34.0 | 12.6 | +21.4 | 7.4E-10 | <0.001 |
| GO:0043232 | intracellular non-membrane-bound organelle | 66 | 34.0 | 12.6 | +21.4 | 7.4E-10 | <0.001 |
| GO:0006412 | translation | 86 | 39.0 | 16.5 | +22.5 | 4.3E-9 | <0.001 |
| GO:0006091 | generation of precursor metabolites and ene... | 69 | 33.0 | 13.2 | +19.8 | 1.6E-8 | <0.001 |
| GO:0005737 | cytoplasm | 260 | 81.0 | 49.8 | +31.2 | 2.0E-8 | <0.001 |
| GO:0005622 | intracellular | 302 | 90.0 | 57.9 | +32.1 | 2.3E-8 | <0.001 |
| GO:0015078 | hydrogen ion transmembrane transporter ac... | 15 | 13.0 | 2.9 | +10.1 | 2.4E-8 | <0.001 |
| GO:0006732 | coenzyme metabolic process | 43 | 24.0 | 8.2 | +15.8 | 4.3E-8 | <0.001 |
| GO:0044464 | cell part | 578 | 142.0 | 110.7 | +31.3 | 4.3E-8 | <0.001 |
| GO:0009059 | macromolecule biosynthetic process | 137 | 51.0 | 26.2 | +24.8 | 5.1E-8 | <0.001 |
| GO:0051186 | cofactor metabolic process | 53 | 27.0 | 10.2 | +16.8 | 7.5E-8 | <0.001 |
| GO:0015077 | monovalent inorganic cation transmembran... | 16 | 13.0 | 3.1 | +9.9 | 1.1E-7 | <0.001 |
| GO:0044237 | cellular metabolic process | 487 | 124.0 | 93.3 | +30.7 | 2.5E-7 | <0.001 |
| GO:0003723 | RNA binding | 72 | 32.0 | 13.8 | +18.2 | 2.6E-7 | <0.001 |
| GO:0009060 | aerobic respiration | 17 | 13.0 | 3.3 | +9.7 | 3.7E-7 | <0.001 |
| GO:0009201 | ribonucleoside triphosphate biosynthetic pro... | 11 | 10.0 | 2.1 | +7.9 | 5.0E-7 | <0.001 |
| GO:0009206 | purine ribonucleoside triphosphate biosynth... | 11 | 10.0 | 2.1 | +7.9 | 5.0E-7 | <0.001 |
| GO:0009145 | purine nucleoside triphosphate biosynthetic ... | 11 | 10.0 | 2.1 | +7.9 | 5.0E-7 | <0.001 |
| GO:0009142 | nucleoside triphosphate biosynthetic process | 11 | 10.0 | 2.1 | +7.9 | 5.0E-7 | <0.001 |
| GO:0022890 | inorganic cation transmembrane transporter... | 22 | 15.0 | 4.2 | +10.8 | 5.0E-7 | <0.001 |
| GO:0044260 | cellular macromolecule metabolic process | 170 | 57.0 | 32.6 | +24.4 | 5.1E-7 | <0.001 |
| GO:0006818 | hydrogen transport | 13 | 11.0 | 2.5 | +8.5 | 5.4E-7 | <0.001 |
| GO:0009141 | nucleoside triphosphate metabolic process | 13 | 11.0 | 2.5 | +8.5 | 5.4E-7 | <0.001 |
| GO:0015992 | proton transport | 13 | 11.0 | 2.5 | +8.5 | 5.4E-7 | <0.001 |
| GO:0044267 | cellular protein metabolic process | 132 | 47.0 | 25.3 | +21.7 | 1.0E-6 | <0.001 |
| GO:0019538 | protein metabolic process | 132 | 47.0 | 25.3 | +21.7 | 1.0E-6 | <0.001 |
| GO:0044238 | primary metabolic process | 459 | 117.0 | 87.9 | +29.1 | 1.1E-6 | <0.001 |
| GO:0045333 | cellular respiration | 18 | 13.0 | 3.4 | +9.6 | 1.1E-6 | <0.001 |

# Split 7 - Low

| Category ID | Category Name | #Genes Split in | #Genes on Path | #Genes Expected | #Genes Enriched | p-value | Corrected p-value |
|---|---|---|---|---|---|---|---|
| GO:0006412 | translation | 47 | 30.0 | 17.0 | +13.0 | 6.1E-5 | 0.006 |
| GO:0003735 | structural constituent of ribosome | 21 | 16.0 | 7.6 | +8.4 | 1.7E-4 | 0.008 |
| GO:0009059 | macromolecule biosynthetic process | 86 | 46.0 | 31.0 | +15.0 | 3.5E-4 | 0.022 |
| GO:0005840 | ribosome | 22 | 16.0 | 7.9 | +8.1 | 4.3E-4 | 0.026 |
| GO:0030529 | ribonucleoprotein complex | 23 | 16.0 | 8.3 | +7.7 | 9.6E-4 | 0.074 |

# Split 9 - Low

| Category ID | Category Name | #Genes Split in | #Genes on Path | #Genes Expected | #Genes Enriched | p-value | Corrected p-value |
|---|---|---|---|---|---|---|---|
| GO:0045333 | cellular respiration | 13 | 12.0 | 4.4 | +7.6 | 1.1E-5 | <0.001 |
| GO:0009060 | aerobic respiration | 13 | 12.0 | 4.4 | +7.6 | 1.1E-5 | <0.001 |
| GO:0015980 | energy derivation by oxidation of organic com... | 13 | 12.0 | 4.4 | +7.6 | 1.1E-5 | <0.001 |
| GO:0009109 | coenzyme catabolic process | 12 | 11.0 | 4.1 | +6.9 | 3.3E-5 | <0.001 |
| GO:0051187 | cofactor catabolic process | 12 | 11.0 | 4.1 | +6.9 | 3.3E-5 | <0.001 |
| GO:0006099 | tricarboxylic acid cycle | 12 | 11.0 | 4.1 | +6.9 | 3.3E-5 | <0.001 |
| GO:0006084 | acetyl-CoA metabolic process | 12 | 11.0 | 4.1 | +6.9 | 3.3E-5 | <0.001 |
| GO:0046356 | acetyl-CoA catabolic process | 12 | 11.0 | 4.1 | +6.9 | 3.3E-5 | <0.001 |
| GO:0046872 | metal ion binding | 30 | 20.0 | 10.2 | +9.8 | 6.8E-5 | <0.001 |
| GO:0043167 | ion binding | 30 | 20.0 | 10.2 | +9.8 | 6.8E-5 | <0.001 |
| GO:0044248 | cellular catabolic process | 20 | 15.0 | 6.8 | +8.2 | 9.1E-5 | 0.002 |
| GO:0006091 | generation of precursor metabolites and ene... | 33 | 21.0 | 11.2 | +9.8 | 1.2E-4 | 0.002 |
| GO:0009056 | catabolic process | 21 | 15.0 | 7.2 | +7.8 | 2.3E-4 | 0.004 |
| GO:0006732 | coenzyme metabolic process | 24 | 16.0 | 8.2 | +7.8 | 4.9E-4 | 0.014 |
| GO:0043169 | cation binding | 18 | 13.0 | 6.1 | +6.9 | 5.9E-4 | 0.014 |
| GO:0051186 | cofactor metabolic process | 27 | 17.0 | 9.2 | +7.8 | 8.6E-4 | 0.020 |
| GO:0046914 | transition metal ion binding | 17 | 12.0 | 5.8 | +6.2 | 1.4E-3 | 0.046 |

# 3. Dynamic Regulatory Maps at Different Score Thresholds

The following two maps are based on the same input data as the maps shown in Figure 5A and 5C of the main manuscript, but with a stricter score requirement of ($10^{-5}$) for a TF and regulatory mode label (1 activator; -1 repressor) to appear on a path out of a split. The split score is computed based on the hypergeometric distribution (see Supporting Methods).

**Map using Prediction Extended Input ($10^{-5}$ Score Threshold)**

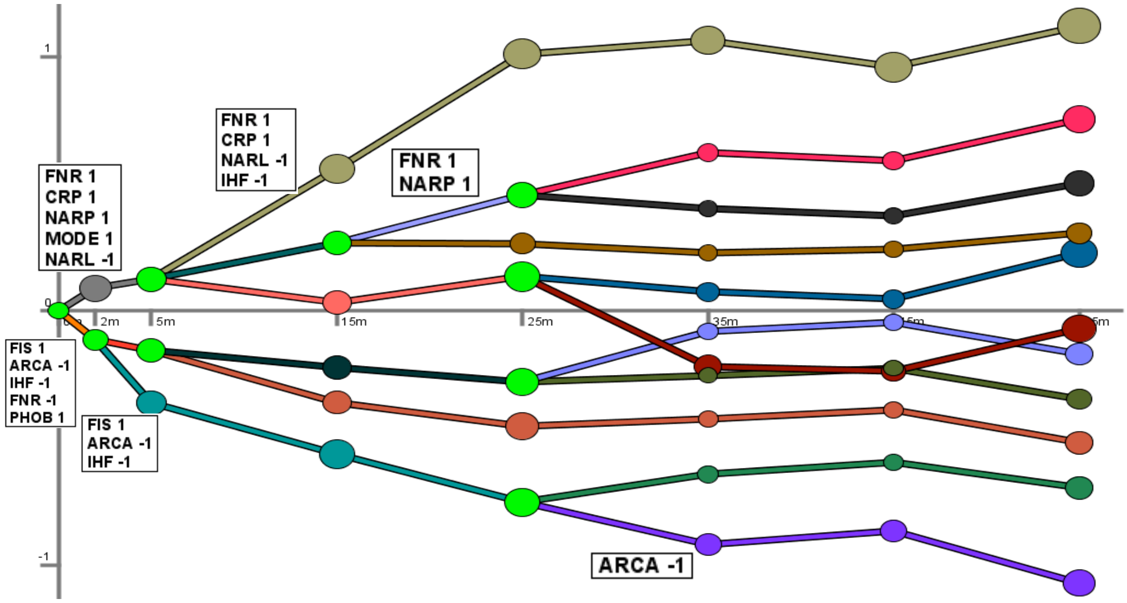


**Map using Curated Input ($10^{-5}$ Score Threshold)**

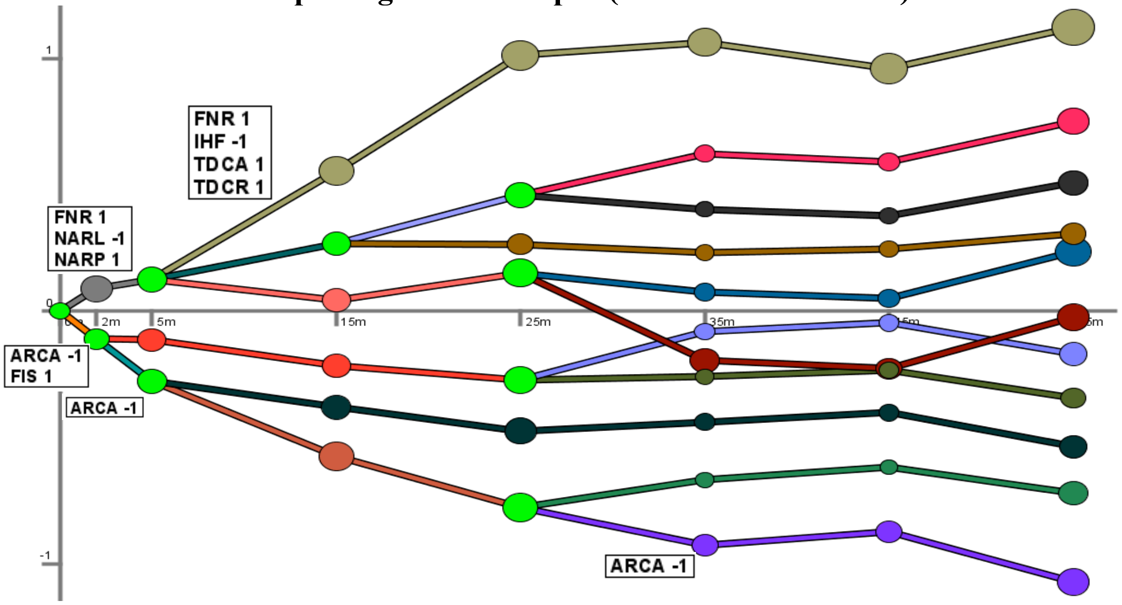

The following maps are based on the same input data as the maps shown in Figure 5A and 5C of the main text, but with a looser score requirement of ($10^{-3}$) for a TF label to appear on a path out of a split.

**Map using Prediction Extended Input ($10^{-3}$ Score Threshold)**

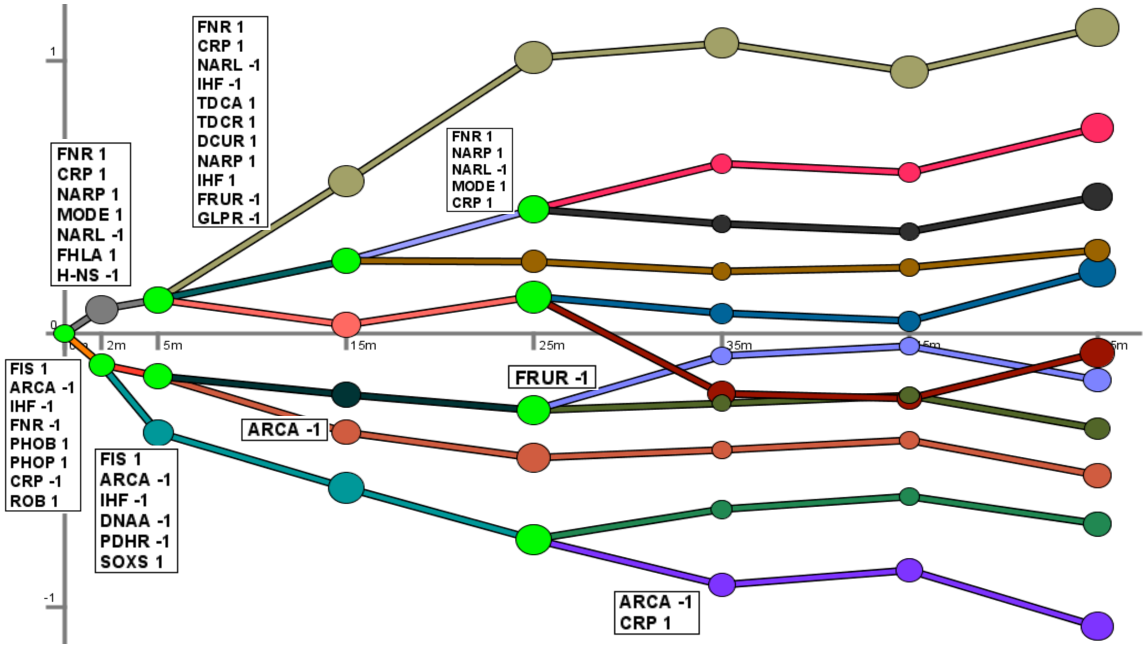


**Map using Curated Input ($10^{-3}$ Score Threshold)**

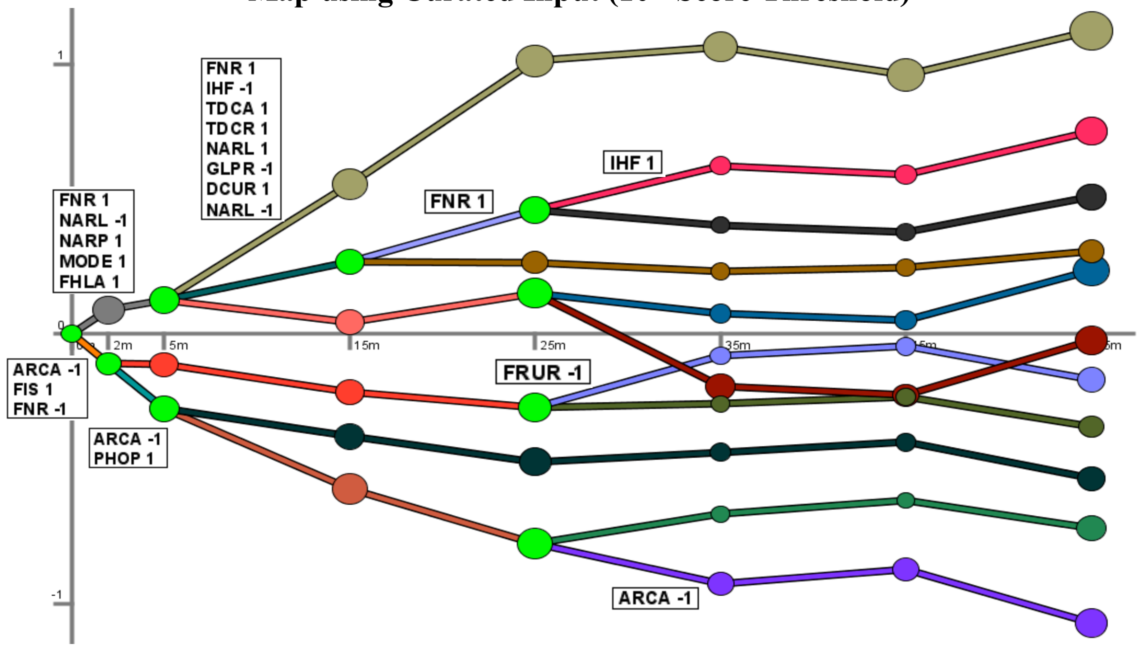

## 4. Motif Scanning Distribution of Location of Sites

For the 71 TFs with a confirmed target based on direct evidence in EcoCyc [5] and a motif positional weight matrix in RegulonDB, we scanned for binding site motifs on both strands in a 350 base pair region around any gene which was the first gene transcribed in a transcriptional unit based on the RegulonDB list [6]. The 350 base pair region ranged from 50 base pairs downstream of the start of the coding sequence to within 300 base pairs upstream of the start of the coding sequence. The base that was closest to the downstream end of the region determined the position of the motif. Only one base of the motif needed to lie within the upstream boundary, but the entire motif would need to be within the downstream boundary. For a gene we associated its motif score with the score in the region of the first gene transcribed in its transcriptional unit. If the gene belonged to several transcriptional units with different initial genes we used the highest score. Below we plot distribution of the location of highest scoring sites we associated with genes, only counting sites with a positive score and a site once for each TF. The plot is aggregated over all 71 TFs. As can be seen the number of motif hits tended to be lower near the boundaries of the region we consider. One can also observe a positional bias for binding sites immediately upstream of the start of the coding region.

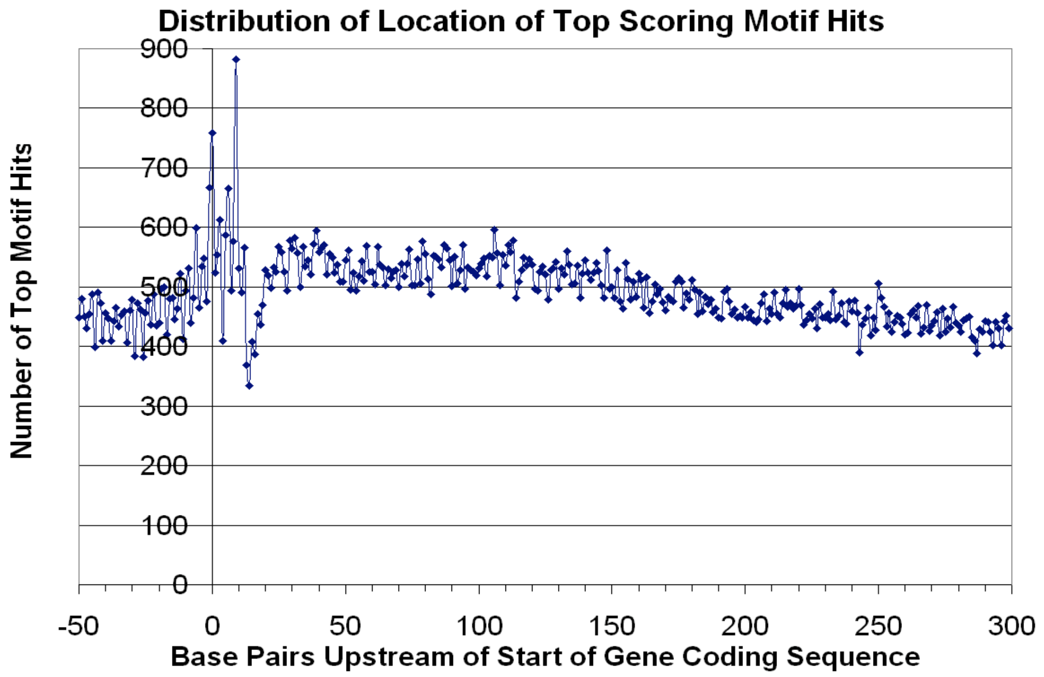

## 5. Extended Analysis of ChIP-chip Validation

In this section, we present below an extended version of Figure 3 from the main manuscript. In the figure below we have added the following to Figure 3:

1. To all the graphs we added a curve which represents the expected 95$^{th}$ percentile performance of a method that randomly orders genes. This curve was computed based on the hypergeometric distribution.
2. For all graphs we added a curve based on using SEREND on only the expression data and direct targets in EcoCyc without the motif input, and also without using the self-training procedure. The values from this curve corresponds to the expression feature of the meta-classifier during the initial iteration of SEREND, before any labels have changed.
3. For all graphs we added a curve based on the results of the Relevance Network approach [7] using the square of the correlation coefficient which was previously reported to outperform mutual information for network inference on this data [8]. We applied the correlation coefficient on the same normalized and transformed gene expression data as described in the main manuscript.
4. For FNR and CRP we added curves based on the direct set of results returned by RegTransBase [9] without extending the list to include other genes listed in the same transcriptional unit.
5. For FNR case we have add a point corresponding to the set of genes listed in the Supplement of Ref. [10] as being differentially expressed in FNR knockouts.
6. For the H-NS case we extend here the list of the H-NS bound targets listed in Ref. [11] to include any other gene in the same RegulonDB [6] transcriptional unit as a gene from this H-NS bound list.

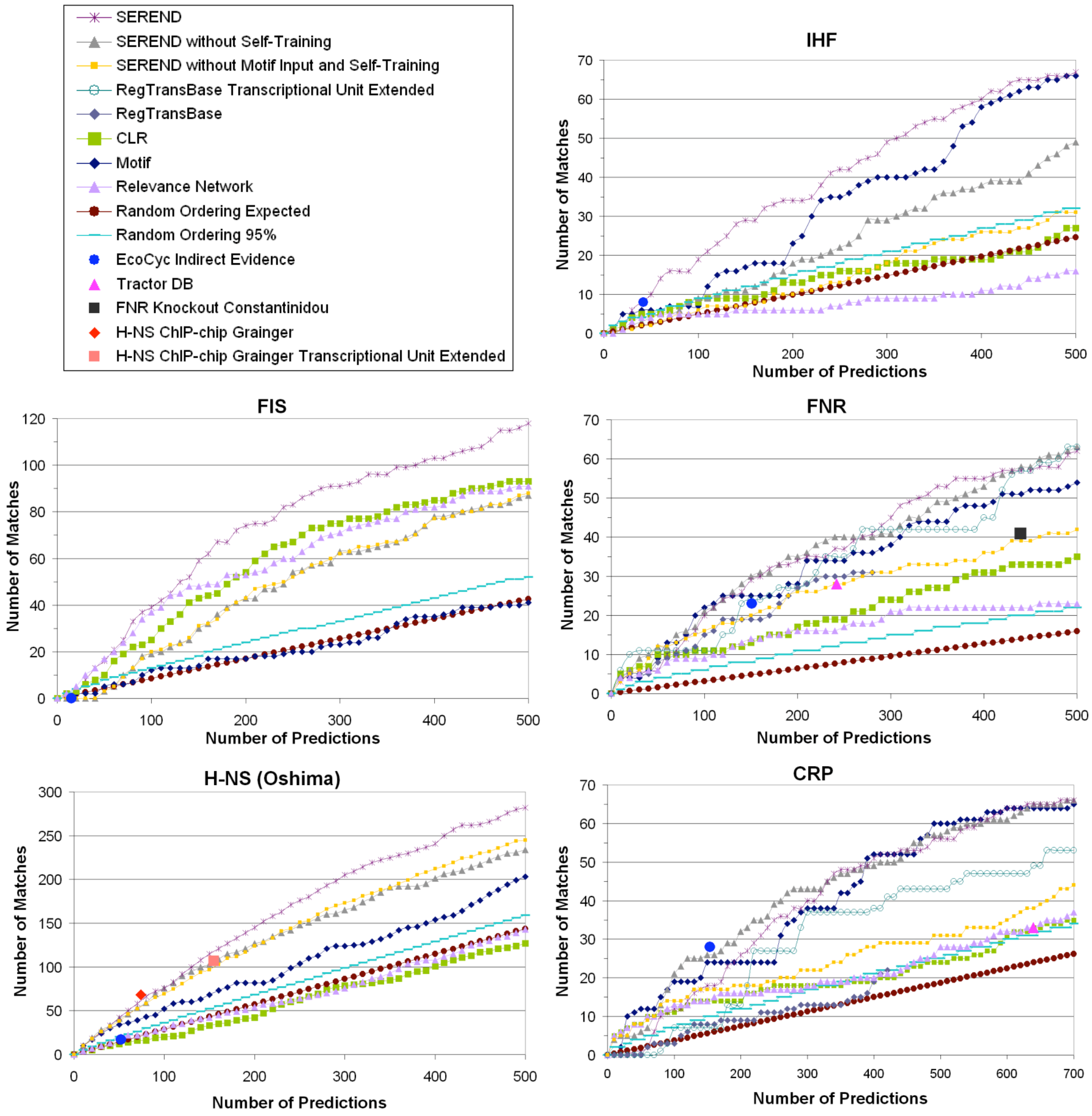
SEREND
SEREND without Self-Training
SEREND without Motif Input and Self-Training
RegTransBase Transcriptional Unit Extended
RegTransBase
CLR
Motif
Relevance Network
Random Ordering Expected
Random Ordering 95%
EcoCyc Indirect Evidence
Tractor DB
FNR Knockout Constantinidou
H-NS ChIP-chip Grainger
H-NS ChIP-chip Grainger Transcriptional Unit Extended
IHF
FIS
FNR
H-NS (Oshima)
CRP
Number of Matches
Number of Predictions

## 6. Effect of Self-Training Parameter *k*

For the self-training procedure as stated in the main text of the manuscript, SEREND would change the label of a gene *g* based on a formula that depends on a parameter *k*. As *k* increases the self-training method requires greater confidence from the classifier that the gene is actually regulated by the TF before changing its label. When *k* is sufficiently high no gene will be relabeled as a target of the TF making the SEREND method similar to a version that does not use self-training.

In the figure below we investigate the effect of this choice of parameter value on the results that would be obtained with our ChIP-chip validation for three more values of *k*: 1.5, 4, and 10. We observe from these figures that in the FNR case with values of *k*=4 and *k*=10 we observe an improvement using self-training that we did not observed when *k*=2. However in the IHF case, the results with *k*=4 and *k*=10 are similar to the results when not using self-training, while there is an improvement over self-training for *k*=1.5 and *k*=2.

## Analysis on the Effect of the Choice of *k* on Recovering ChIP-chip targets without Direct Evidence

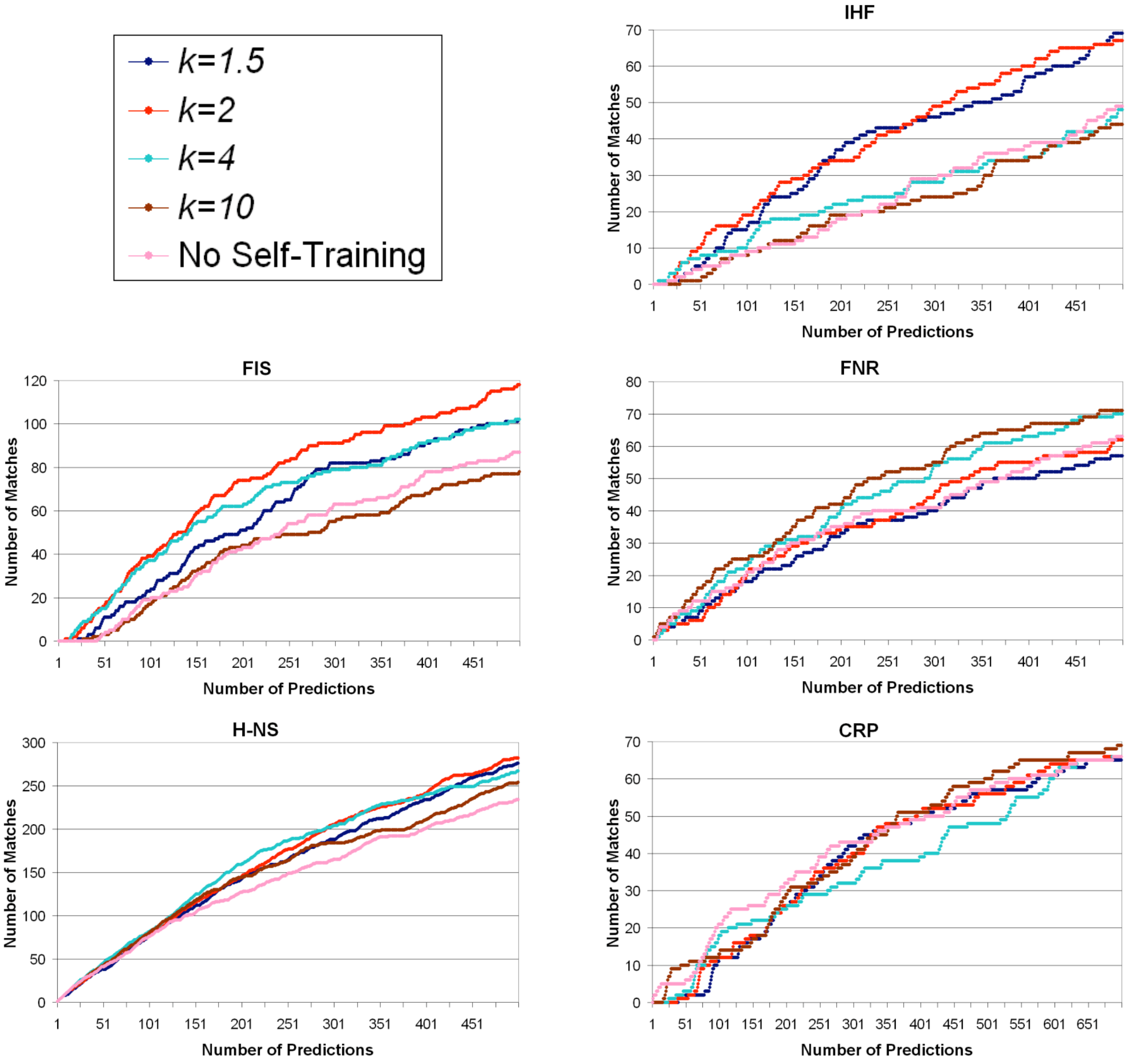

## 7. Effect of Randomly Extending the Curated Network

In Figure 6A of the main manuscript, we showed that by extending the curated inputs with additional predictions DREM increased the score significance of most of the key TFs. To investigate whether this could be a result of simply predicting more TF-gene interactions we generated 10,000 sets of randomly extended TF-gene inputs. These randomly extended inputs had the same curated targets as the prediction extended network, additionally among the genes used in the aerobic-anaerobic shift application of DREM we also constrained a random extension to have the same number of predicted targets for each TF and regulatory mode (activator or repressor) as in our prediction extended network. For those genes that were a target of a TF in both the curated and prediction extended network, but had a different regulatory mode annotation, we used the annotation in the prediction extended input. The DREM software [12] contains the option to either use TF-gene input in inferring the model or only as a post-processing step. If the TF-gene input was used to infer the model, then the transition probabilities for a gene between states were based on the specific set of TFs regulating the genes as determined based on an Input-Output Hidden Markov Model (IOHMM). If the option to use TF-gene input only in a post-processing step is selected, then DREM infers the regulatory map using only the time series expression data, and under this option the transition probabilities are the same for each gene and the model is an instance of a Hidden Markov Model (HMM). For both the IOHMM and HMM, the TF-gene inputs were used in a post-processing step to score TFs at splits. While we use the IOHMM to infer the dynamic maps presented in this paper, we observed for this data that the difference in scores given to TFs using the HMM and IOHMM model for both the curated and prediction extended input was minor compared to the difference in scores based on the differences of these input sets (see below). Thus in our evaluation we only inferred a model once based on the time series data using an HMM, which we then in a post-processing step scored using all 10,000 randomly extended input sets sets. As can be seen in the figure below even the $95^{th}$ percentile of scores resulting from these randomly extended inputs did not improve over just using the curated inputs.

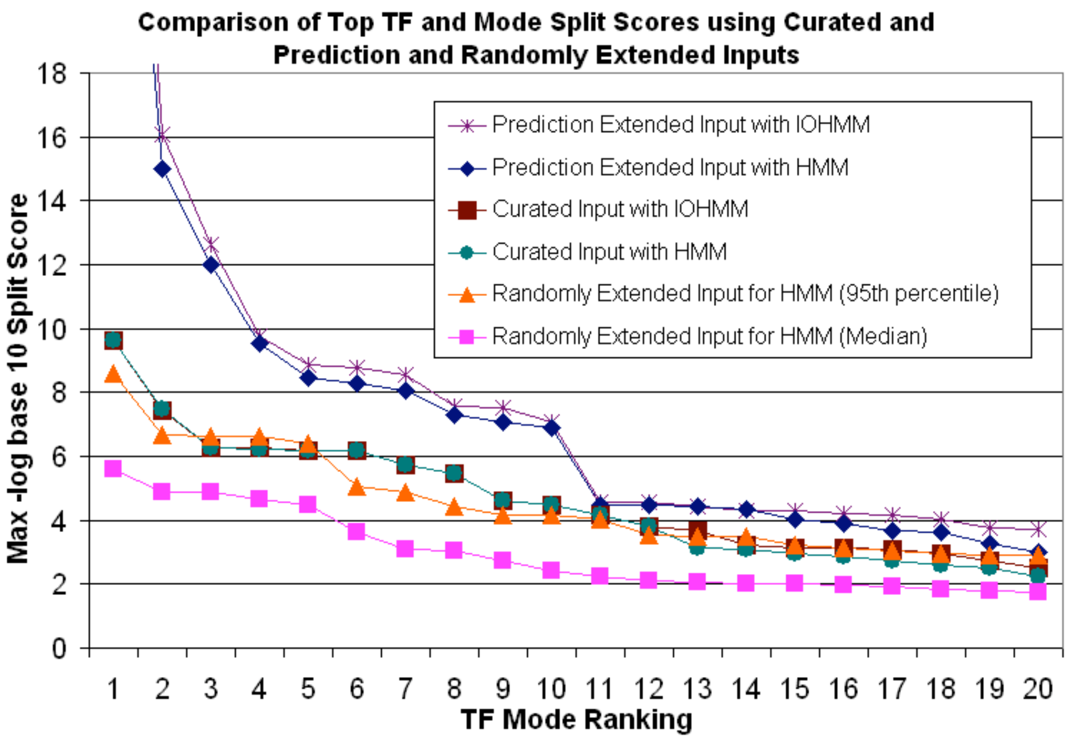


The graph shows the top 20 values of the maximum negative log base 10 scores for a TF and mode (activator or repressor) at any split, only including a TF and mode pair once. We evaluated this for the curated input, as well as the random and prediction extended inputs using a HMM, and also for the curated input and prediction extended input using an IOHMM. To keep the scale of the graph reasonable the highest score with the prediction extended input using an IOHMM (38.2) and using an HMM (33.4) are not shown in the figure.

We also provide in the figure below a dynamic map using the IOHMM model and the first input set that was randomly generated based on a scoring threshold of $10^{-4}$. We note that this map has fewer TF-labels at an equivalent scoring threshold than, both the curated input and the prediction extended input shown in Figure 5 of the main text, which were based on the same scoring threshold.

**Map using a Randomly Extended Input Set ($10^{-4}$ Score Threshold)**

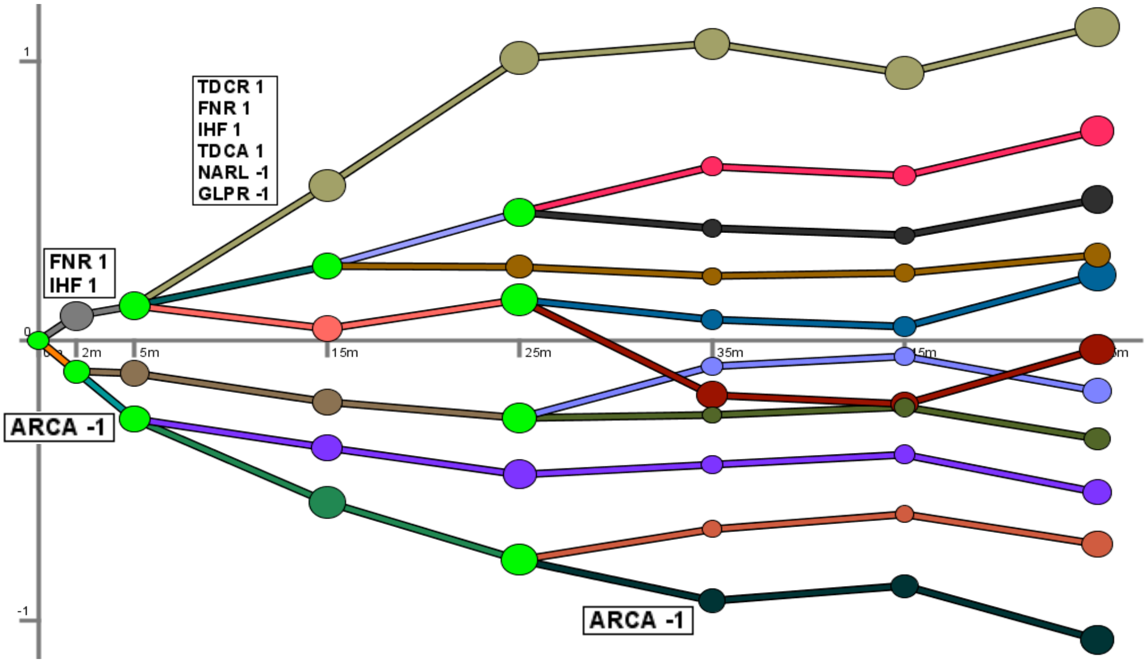

# References

1. Kang Y, Weber KD, Qiu Y, Kiley PJ, Blattner FR (2005) Genome-wide expression analysis indicates that FNR of *Escherichia coli* K-12 regulates a large number of genes of unknown function. J Bacteriol 187: 1135–1160.

2. Salmon K, Hung SP, Mekjian K, Baldi P, Hatfield GW, et al. (2003) Global gene expression profiling in *Escherichia coli* K12. The effects of oxygen availability and FNR. J Biol Chem 278: 29837–29855.

3. Salmon K, Hung SP, Steffen NR, Krupp R, Baldi P, et al. (2005) Global gene expression profiling in *Escherichia coli* K12: effects of oxygen availability and ArcA. J Biol Chem 280: 15084–15096.

4. Shalel-Levanon S, San KY, Bennett GN (2005) Effect of ArcA and FNR on the expression of genes related to the oxygen regulation and the glycolysis pathway in *Escherichia coli* under microaerobic growth conditions. Biotechnol Bioeng 92: 147-159.

5. Keseler IM, Collado-Vides J, Gama-Castro S, Ingraham J, Paley S, et al. (2005) EcoCyc: A comprehensive database resource for *Escherichia coli*. Nucleic Acids Res 33: D334-337.

6. Salgado H, Gama-Castro S, Peralta-Gil M, Diaz-Peredo E, Sanchez-Solano F, et al. (2006) RegulonDB (version 5.0): *Escherichia coli* K-12 transcriptional regulatory network, operon organization, and growth conditions. Nucleic Acids Res 34: D394-397.

7. Butte AJ, Tamayo P, Slonim D, Golub TR, Kohane IS (2000) Discovering functional relationships between RNA expression and chemotherapeutic susceptibility using relevance networks. Proc Natl Acad Sci U S A 97: 12182–12186.

8. Faith JJ, Hayete B, Thaden JT, Mogno I, Wierzbowski J, et al. (2007) Large-scale mapping and validation of *Escherichia coli* transcriptional regulation from a compendium of expression profiles. PLoS Biology 5: e8.

9. Kazakov AE, Cipriano MJ, Novichkov PS, Minovitsky S, Vinogradov DV et al. (2007) RegTransBase-a database of regulatory sequences and interactions in a wide range of prokaryotic genomes. Nucleic Acids Res 35: D407-D412.

10. Constantinidou, C, Hobman, JL, Griffiths L, Patel, MD, Penn CW, et al. (2006) A reassessment of the FNR regulon and transcriptomic analysis of the effects of nitrate, nitrite, NarXL, and NarQP as *Escherichia coli* K12 adapts from aerobic to anaerobic growth. J Biol Chem 281: 4802-4815.

11. Grainger DC, Aiba H, Hurd D, Browning DF, Busby SJW (2007) Transcription factor distribution in *Escherichia coli*: studies with FNR protein. Nucleic Acids Res 35: 269-278.

12. Ernst J, Vainas O, Harbison CT, Simon I, and Bar-Joseph Z (2007) Reconstructing dynamic regulatory maps. Mol Syst Biol 3: 74.
