# Supplementary material for: A Semi-Supervised Method for Predicting Transcription Factor–Gene Interactions in Escherichia coli
Source: PLoS Comput Biol. 2008 Mar 28;4(3):e1000044. doi: 10.1371/journal.pcbi.1000044 (PMC2266799; doi:10.1371/journal.pcbi.1000044)
Supplement: Text S2 Supporting Methods — (0.19 MB PDF) [file pcbi.1000044.s002.pdf]

Supporting Methods for:

A Semi-Supervised Method for Predicting Transcription Factor-Gene Interactions in *Escherichia coli*

Jason Ernst[1], Qasim K. Beg[2,3], Krin A. Kay[2], Gábor Balázsi[4], Zoltán N. Oltvai[2], Ziv Bar-Joseph[1]

[1]Machine Learning Department, School of Computer Science, Carnegie Mellon University, Pittsburgh, PA, 15213, USA

[2]Department of Pathology, University of Pittsburgh, Pittsburgh, PA, 15261, USA

[4]Department of Systems Biology, University of Texas M. D. Anderson Cancer Center, Houston, TX, 77054, USA

[3]Current Address: Department of Biomedical Engineering, Boston University, Boston, MA, 02215, USA

Contents

1. Dynamic Regulatory Event Maps p2-3

2. Microarray Data Preprocessing p4-5

References: p6

1. Dynamic Regulatory Event Maps

The dynamic regulatory event maps were inferred from time series expression data and transcription factor (TF)-gene interaction input using the Dynamic Regulatory Events Miner (DREM) [1]. The TF-gene interaction input was a grid, such that for any pair of TF and gene, there was a '1' if the TF was considered primarily an activator of the gene in the input, '-1' if the TF was primarily a repressor of the gene, and '0' if it did not regulate the gene. The maps correspond to a probabilistic model as described in Ref. [1], which we briefly review here. Each node in the map corresponds to a state associated with a Gaussian emission distribution for the expression levels of the genes going through that state. The edges correspond to the valid transition between states in the model. The set of valid transitions was set to enforce a tree structure among the states with each state having at least one possible transition to a state at the next time point and at most three possible transitions. If a gene goes through a state for which there were multiple transitions out of the state, the state to which a gene would transition to next was modeled probabilistically. The transition probabilities would depend on the TF-gene interaction data. Logistic regression classifiers were used to map the static TF-gene interaction input for a gene to the transition probabilities (note that these transition classifiers are separate classifiers from those used to predict new TF-gene interactions as part of SEREND). The model selection procedure which determines the number of states and the constraints on the valid transitions was simplified from the method used in the work of Ref. [1]. In that work a subset of the genes was used to train the parameters of the model, and the remaining genes was used when selecting the model structure. Here we used all the data to train the parameters and select the model structure. A regularization penalty on the number of states was used to prevent overfitting the data. The model which maximized the following expression was selected:

$$\log(L(X \mid \Theta)) - \eta NumberStates(\Theta)$$

where X was the data, Θ was the model structure and parameter settings, $L(X \mid \Theta)$ was the likelihood of the data given the model structure and parameter settings in the model, and $NumberStates(\Theta)$ was the number of states in the model. Finally η was a regularization parameter that was set to 40.

Once a final model was selected, genes were assigned to their most likely path through the model based on the expression of the genes and the set of TF-gene interactions for the gene. A TF with its regulation mode (activator or repressor) was associated with a path out of a split if its association score was below a specified threshold, with a lower score being more significant. The association score for a specific TF and regulation mode on a path out of a split was computed using the hypergeometric distribution as

$$\sum_{i=k}^{\min(m,n)} \frac{\binom{m}{i}\binom{N-m}{n-i}}{\binom{N}{n}}$$

where *N* is total number of genes going into the split, *m* is the number of genes going into the split regulated by the TF in the specified regulation mode, *k* is the number of genes assigned to the path and were regulated by the TF in the specified regulation mode, and *n* the total number of genes on the path.

2. Microarray Data Preprocessing

Two-color cDNA microarray data are never devoid of spurious technical contributions that originate during array printing, as well as during the collection and processing of samples, fluorescent labeling and hybridization and scanning of the microarray images [2]. To minimize the effect of such contributions, microarray data were normalized as described before [3].

Briefly, we excluded spots from further analysis if the foreground intensity of less than 50% of the pixels within the spot were above 2 standard deviations of the background. We generated expression data tables in Microsoft Excel, containing the following information for each of the 14,352 entries: block (B), column (X), and row (Y) number, red foreground (f_r) and background (b_r), green foreground (f_g) and background (b_g) intensity. The position of each probe within a block, P is defined by the pair of integers (X,Y).

Log-ratios were defined as the base-10 logarithm of $(f_r–b_r)/(f_g–b_g)$, where f_r, b_r, f_g and b_g represent the median Cy5 (red) foreground and background, and the median Cy3 (green) foreground and background intensities, respectively. In some cases, when the intensity of the background was higher than or equal to the intensity of the foreground, the resulting log-ratios became complex or infinity. We eliminated these values using the find, imag, and isfinite functions in Matlab.

Next, we normalized data by averaging the log-ratios resulting from all spots printed by a print tip, and subtracting the resulting average from all the individual log-ratios corresponding to the same tip. We averaged and listed in a new file all the log-ratios of the same gene from each slide. If a repeat slide was available for a time point (5m, 25m, and 55m) then we averaged the values. We eliminated about two dozen outlier values that were not reproduced across slides if a repeat time point was available, or across the multiples spots for the gene within one slide.

We transformed all values to the difference with their 0 min experiment value. If the 0 min value was missing, then we first imputed its value based on its 2 min value. We did this imputation by first grouping genes that had both 0 min and 2 min values based on the 2 min value. The groups were based on non-overlapping intervals of length 0.1 in log

base 10 except we had a group for all values greater than 0.4 and a group for less than -0.4. We computed the average 0 min value for genes in each group and used this for the imputation. If both the time point 0 min and 2 min were eliminated then we used a value of zero for the 0 min time point. Finally we filtered spots that did not have a Blattner number associated with them from further analysis. For the DREM analysis genes were further filtered if all values were eliminated for two or more time points other than the 0m time point, or there was not an absolute log base 10 change of at least 0.3 at any time point.

References

1. Ernst J, Vainas O, Harbison CT, Simon I, and Bar-Joseph Z (2007) Reconstructing dynamic regulatory maps. Mol Syst Biol 3: 74.

2. Balázsi G, Kay KA, Barabási AL, Oltvai ZN (2003) Spurious spatial periodicity of co-expression in microarray data due to printing design. Nucleic Acid Res 15: 4425-4433.

3. Tong X, Campbell JW, Balázsi G, Kay KA, Wanner BL, et al. (2004) Genome-scale identification of conditionally essential genes in *E. coli* by DNA microarrays. Biochem Biophys Res Commun 322: 347-354.
